# Pericentromeric Satellite III transcripts induce etoposide resistance

**DOI:** 10.1038/s41419-021-03810-9

**Published:** 2021-05-24

**Authors:** Julian Kanne, Michelle Hussong, Jörg Isensee, Álvaro Muñoz-López, Jan Wolffgramm, Felix Heß, Christina Grimm, Sergey Bessonov, Lydia Meder, Jie Wang, H. Christian Reinhardt, Margarete Odenthal, Tim Hucho, Reinhard Büttner, Daniel Summerer, Michal R. Schweiger

**Affiliations:** 1 Institute for Translational Epigenetics, University Hospital of Cologne, Faculty of Medicine, University of Cologne, Cologne, Germany; 2 Center for Molecular Medicine Cologne, University of Cologne, Cologne, Germany; 3 Translational Pain Research, Department of Anaesthesiology and Intensive Care Medicine, University Hospital Cologne, Faculty of Medicine, University Cologne, Cologne, Germany; 4 Faculty of Chemistry and Chemical Biology, TU Dortmund University, Dortmund, Germany; 5 Rheinische Fachhochschule Cologne, Cologne, Germany; 6 Department I of Internal Medicine, University Hospital Cologne, Medical Faculty, Cologne, Germany; 7 Institute of Pathology, University Hospital of Cologne, Medical Faculty, Cologne, Germany; 8 Department of Hematology and Stem Cell Transplantation, University Hospital Essen, University Duisburg-Essen, German Cancer Consortium (DKTK partner site Essen), Essen, Germany; 9 Center of Integrated Oncology Cologne-Bonn, Medical Faculty, University of Cologne, Cologne, Germany

**Keywords:** Cancer therapeutic resistance, Epigenetics, Long non-coding RNAs

## Abstract

Non-coding RNA from pericentromeric satellite repeats are involved in stress-dependent splicing processes, maintenance of heterochromatin, and are required to protect genome stability. Here we show that the long non-coding satellite III RNA (SatIII) generates resistance against the topoisomerase IIa (TOP2A) inhibitor etoposide in lung cancer. Because heat shock conditions (HS) protect cells against the toxicity of etoposide, and SatIII is significantly induced under HS, we hypothesized that the protective effect could be traced back to SatIII. Using genome methylation profiles of patient-derived xenograft mouse models we show that the epigenetic modification of the *SatIII* DNA locus and the resulting SatIII expression predict chemotherapy resistance. In response to stress, SatIII recruits TOP2A to nuclear stress bodies, which protects TOP2A from a complex formation with etoposide and results in decreased DNA damage after treatment. We show that BRD4 inhibitors reduce the expression of SatIII, restoring etoposide sensitivity.

## Introduction

Pericentromeric satellite RNAs are transcribed from heterochromatic regions under cellular stress conditions. The advancement in high-throughput technologies allows to map and characterize non-protein coding (NPC) regions of the genome and has resulted in the discovery that heterochromatin is not generally transcriptionally inert, but plays versatile roles in development and disease^1^. NPC regions are divided into non-repetitive and repetitive regions. A major subgroup of repetitive regions are the tandem repetitive DNAs, which include the prominent repeats: telomeric DNAs, microsatellites, and satellites^2^. Satellites are predominantly located at centromeric and pericentromeric regions of chromosomes and mediate the faithful distribution of the duplicated genome to daughter cells. Heterochromatic satellite DNAs are structurally characterized by repressive histone marks (H3K9me2/3, H4K20me2/3), a high degree of DNA methylation, and the presence of heterochromatin protein 1 (HP1)^3^. Upon relaxation of heterochromatin, and especially following loss of H3K9me2, satellite RNA transcripts are derepressed what leads to RNA:DNA loop formation, the accumulation of R-loops, and excessive strand breakage and DNA damage^4,5^. Similar to histone methylation, the BRCA1 complex protects against replication defects, satellite repeat transcription and RNA:DNA hybrid formation^6,7^. Though the complex organization of repetitive regions is difficult to grasp, a thorough understanding of errant stress responses in light of heterochromatin relaxation is essential to uncover new mechanisms of disease development.

Satellite RNA transcription can be induced by an array of external triggers, most prominently by heat stress (HS) that results in transcriptional induction of SatIII and an accumulation of these transcripts at their genomic loci, which ultimately leads to the formation of nuclear stress bodies (nSBs)^8–11^. The function of these subnuclear, membrane-less structures and pericentromeric transcripts is largely unknown. Several transcriptional regulators, such as CREB (cAMP response element-binding protein)-binding protein (CREBBP), Pol II, and the heat shock factor 1 (HSF1) are found at nSBs^9,10^. Stress-induced splicing processes are modulated by the recruitment of splicing factors at nSBs^12^. We previously identified the epigenetic regulator BRD4 (Bromodomain protein 4) as an additional component of nSBs^13^ and showed that recruitment of BRD4 to the pericentromeric regions influences the stress-induced splicing process and the heat-mediated induction of SatIII RNA.

Though the loss of heterochromatin and subsequent transcription of non-coding RNAs from satellite repeats is induced in mouse and human epithelial cancers, including pancreatic, colon, and lung tumors^14,15^, *SatIII* repeats have not been reported to have therapeutic relevance. HS conditions protect cells against the toxicity of chemotherapeutic drugs, most prominently the topoisomerase 2 (TOP2) inhibitor etoposide^16^. Because SatIII is significantly induced under HS, we hypothesized that the protective effect could be traced back to SatIII. Etoposide treatment is part of a broad range of cancer treatment regimens and is frequently used to treat lung cancer. Etoposide temporarily stabilizes transiently induced DNA double-strand breaks (DSB) created by TOP2A. The interaction of etoposide with TOP2A promotes the emergence of stable TOP2A cleavage complexes (TOP2ccs) and causes defective DNA re-ligation and rewinding. This results in DNA damage, which induces the DNA damage response and leads to apoptosis^17–20^. Cellular stress response mechanisms, including DNA damage repair pathways, may counteract this effect and enable therapy resistant cancer cells to evade the toxic effect of etoposide.

We report here that the de-methylation and expression of SatIII in non-small cell lung cancer patient-derived xenograft mouse models (NSCLC-PDX) and cell culture models promote cellular resistance towards etoposide. We show that the recruitment of the etoposide target TOP2A to nSBs is SatIII dependent and results in decreased DNA damage that impacts downstream DNA repair pathways. Etoposide resistance can be overcome by inhibiting SatIII expression by BRD4 inhibitors. Our work identifies the first repetitive non-coding RNA that confers etoposide resistance, as well as proposes that chemically induced alterations in SatIII expression can be utilized to overcome etoposide resistance.

## Materials and methods

### Cell lines and HS conditions

HeLa (ATCC, CCL-2, RRID: CVCL0030), U2OS (ATCC HTB-96, RRID:CVCL0042), H2030 (ATCC CRL-5914, RRID:CVCL1517), and HCC827 (ATCC CRL-2868, RRID:CVCL2063) were purchased from ATCC. HEKT293 (Thermo R70007, RRID: CVCL6911) were purchased from Thermo Scientific. HeLa and U2OS cells were cultivated in Dulbecco’s Modified Eagle’s Medium (Biochrom), containing 10% fetal calf serum, 2 mM L-glutamine, and 100 U penicillin/streptomycin. H2030, HCC827: RPMI 1640 Medium, containing 10% fetal calf serum, 2 mM L-glutamine, and 100 U penicillin/streptomycin. HEK T293: DMEM GlutaMAX™ Medium, containing 10% fetal calf serum and 100 U penicillin/streptomycin. All cell lines were tested negative for mycoplasma contamination. Cell line data were collected from Cancerrxgene (Wellcome Sanger Institute) and RNA-Seq data were obtained from Klijn et al.^21^.

For heat stress induction, cells were incubated at 44 °C with 5% CO_2_. Preliminary experiments in HeLa cells and U2OS cells revealed no substantial difference between 42 °C for 4 h and 44 °C for 1 h on RNA level in our hands^13^. Thus, the latter conditions were applied for subsequent experiments, as they induced SatIII foci in a comparable or even stronger fashion.

### Transfection and viral transduction

Transfections were performed with respective siRNAs (SatIII, Control) using Lipofectamine RNAiMAX reagent (Invitrogen Inc., #13778030) according to the manufacturer’s recommendations. Additionally, a modified antisense oligonucleotide was transfected using Lipofectamine 2000 (Invitrogen Inc., #11668027). Sequences of siRNA/shRNA/antisense-oligos are provided in Supplementary Table 1.

For viral transductions plasmids psPAX2 (Dull et al., 1988, RRID:Addgene_12260), MD2.G (Dull et al., 1988, RRID:Addgene_12259) were used and transfected with PEI (Polysciences, #23966-1), Lentiviruses were harvested after 48 h and used for transductions.

### Patient-derived xenograft (PDX) models

The PDX models used in this work are described in detail in Grasse et al.^22^. In brief, patient lung tumor samples were implanted subcutaneously into 1–3 nude or NOD/SCID mice. For the generation of PDXs, primary NSCLC tumor samples with a tumor cell content ranging from 5% to more than 70% were used. For each PDX model, six mice were exposed to treatments per injection or solvent intraperitoneal at days 1 and 8 and tumor growth was measured by caliper measurement for 2–6 weeks. Once tumors became palpable, tumor size was measured weekly with a caliper-like instrument. Individual tumor volume V was calculated with the following formula: V = 1/2 length × width^2^. Tumors of each model were further transplanted into 2–4 mice after a tumor volume of approx. 1.2 cm^3^ was reached. Where possible, snap-frozen tumor samples from each passage (up to 10 passages) were conserved and stored at − 80 °C for further analysis. Chemosensitivity testing was performed as described before in male NMRI:nu/nu mice^23^. To this end, 6 mice were randomly assigned to each control or treatment group. Treated to control (T/C) values of relative tumor volume were used for the evaluation of the treatment. Methylated immunoprecipitations followed by sequencing (MeDIP-Seq) analyses had been performed from 22 PDX tumors and normal lung tissues and made publicly available in Grasse et al. 2018^22^. This MeDIP-Seq data was used for methylation analyses of repetitive elements.

### Methylation analyses of repetitive elements

For the genome-wide methylation analyses of repetitive elements the RepEnrich2 tool was used^24^. Reads were initially aligned to the unmasked genome using Bowtie2^25^ (RRID:SCR_005476) and divided into uniquely mapped and multi-mapped reads. Uniquely mapping reads were tested for overlap with repetitive elements, while multi-mapped reads were separately aligned to repetitive element assemblies identified with RepEnrich2 tool. The repetitive element assemblies are represented by all genomic instances of an individual repetitive element subfamily. Further differential expression analyses were performed using EdgeR^26^ (R/Bioconductor). The region displayed in Fig. 4B is the region that was assigned by the RepEnrich2 tool for RNA-seq analyses. The region displayed shows a high enrichment of SatIII (which is recruited to its own genomic locus) upon HS.

For visualization the bigwig files were converted to bedgraph files that were used for visualization with R/Bioconductor GViz package ^27^.

### Chromatin immunoprecipitation

HeLa cells were exposed to 44 °C for 1 h and immediately fixed for 10 min with 1% formaldehyde (Carl Roth Inc) at RT followed by 5 min blocking with 125 mM Glycine at RT. Chromatin was extracted using the truChlP Chromatin Shearing Kit (Covaris, #520154) according to the manufacturer´s instructions. The chromatin was sheared by sonication to a DNA fragment size of 200–600 bp and precipitated using an antibody against human BRD4 (Bethyl Laboratories, A301-985A100, RRID:AB_2620184). ChIPs were run on the IP-Star compact system using the Auto iDeal ChIP-seq kit for histones (Diagenode, #C01010051) according to the manufacturer’s recommendations for ChIP preparation. ChIP-DNA was sequenced on a HiSeq4000, 50-bp single-end. Reads were mapped to the hg19 genome using bwa-0.7.12 with default parameters^28^. Peaks were called with MACS2^29^ with the parameters “bw 500 -mfold 2100 -broad broad-cutoff 0.1 –bdg”. For visualization the bigwig files were converted to bedgraph files that were used for visualization with R/Bioconductor GViz package ^30^.

### **RNA immunoprecipitation**

HeLa cells were subjected to three different treatment conditions: HS (1 h, 44 °C), HS with a 24 h recovery time at 37 °C, and non-HS conditions (constantly 37 °C). The cells were fixed for 10 min with 1% formaldehyde (Carl Roth Inc) at RT followed by a 5 min blocking step with glycine 125 µM. The harvested cells were lysed with Farnham Lysis buffer (5 mM PIPES pH 8.0; 85 mM KCl; 0.5% NP-40, 100 U/ml SUPERase (Ambion, AM2694)) at 4 °C. Chromatin was sheared by sonication (Bioruptur, Diagenode) to a DNA fragment size of 200–600. RNA was precipitated using an antibody against human TOP2A (SigmaAldrich, #SAB4502998, RRID:AB_10753226) and HSF1 (Santa Cruz Biotechnology, #sc-17757, RRID:AB_627753). RNA was reversely crosslinked and purified using Trizol (SigmaAldrich) and RNA Micro purification Kit (Zymo). After reverse transcription of the purified RNA, enrichment of SatIII was determined with qPCR.

### **Cell viability assay**

Cell viability was measured using the AlamarBlue® Cell Viability Assay (Life Technologies, #DAL1025) according to the manufacturer’s recommendations. In brief, cells were transfected with the referring oligos and cultured for 24 h. Again 24 h later 10 µL of AlamarBlue® reagent was added. Fluorescence intensities were measured 3 h after the addition of the reagent by Infinite 200 PRO Tecan Microplate Reader (Tecan Germany GmbH).

### **Caspase 3/7 activity assay**

Apoptotic activity was determined using Amplite™ Fluorimetric Caspase 3/7 Assay Kit (AAT Bioquest, #13503) following manufacturer’s instructions. In brief, 24 h before starting the assay, cells were transfected in a 96-well frame with the plasmid constructs/siRNA of interest. The next day, caspase 3/7 assay loading solution was prepared by adding 50 µl Caspase 3/7 Substrate (Component A) into 10 ml Assay Buffer (Component B) and mixing well. Then, 100 µl loading solution was pipetted to the transfected cells and the plate was incubated for at least 1 h at room temperature in the dark. The plate was centrifuged at 800 rpm for 2 min. Fluorescence was measured either at 350 nm excitation and 450 nm emission (for *Blue Fluorescence*) or at 490 nm excitation and 525 nm emission (for *Green Fluorescence*) in the plate reader Infinite 200 PRO.

### **Cell cycle analyses**

To determine the cell cycle stage, the distribution of the total Hoechst intensity values acquired from quantitative high-content screening microscopy data were measured. Four thresholds were adjusted manually to gate cells in 2N (G0/G1), 2-4N (S), and 4N (G2/M) stages, respectively. Gates were applied to evaluate the mean number of foci in cells of the respective cell cycle stage. Single-cell data were further processed and plotted using custom R scripts.

### **Cell proliferation assay**

Cells were treated with either DMSO (1%, SigmaAldrich), etoposide (100 µM, Cayman chemical, CAS 33419-42-0), JQ1 (5 µM, Cayman chemical, 1268524-70-4), and CPI-203 (1 µM, BioCat, CAS 1446144-04-2) and immediately incubated at 37 °C. Two pictures of each well were taken every 30 min for a total of 48 h by ImageXpress Micro4 (Molecular Devices). The pictures were analyzed using Cell Profiler software (Broad Institute, RRID:SCR_007358).

### **Immunoblotting**

Cell lysates were obtained by cell lysis using Pierce lysis buffer (25 mM Tris–HCl pH 7.4, 150 mM NaCl, 1 mM EDTA, 1% NP-40, and 5% glycerol) containing a protease inhibitor cocktail (Roche Diagnostics). The lysates were separated using SDS-PAGE and transferred onto nitrocellulose membranes. The membrane was blocked in 5% skim milk-TBST, incubated with primary antibodies: TOP2A (SigmaAldrich #SAB4502998, RRID:AB_10753226, 1/1000), GAPDH (Invitrogen#AM4300, RRID: AB_2536381), washed three times (10 min each with TBST-0.1%Tween-20) and subsequently incubated with peroxidase-conjugated secondary antibodies. Chemiluminescence was performed using Clarity Western ECL Substrate (BioRad).

### **Immunofluorescence staining and imaging**

For immunofluorescence experiments, cells were plated on glass coverslips in 24-well plates and treated as described above. Cells were washed with phosphate buffered saline (PBS, Biochrom) and fixed with 1% formaldehyde (Carl Roth Inc.) for 10 min. Afterwards, cells were washed with PBS and blocked with 0.5% bovine serum albumin (BSA, Sigma) in PBS, containing 0.03% Triton X-100) for 1 h. Cells were incubated with the respective primary antibodies in 0.5% BSA/PBS overnight at 4 °C: BRD4 (Abcam, #ab75898 RRID:AB_1860650, 1/200), 53BP1 (Merck Millipore, #MAB3802, RRID:AB_2206767, 1/400), TOP2A (SigmaAldrich, #SAB4502998, RRID:AB_10753226, 1/250). Cells were then incubated with secondary antibodies for 1 h at room temperature: goat anti-mouse Alexa Fluor 488, goat anti-rabbit Alexa Fluor 488, goat anti-mouse Alexa Fluor 594 and goat anti-rabbit Alexa Fluor 594 (Molecular Probes, #A11029, RRID:AB_138404; #R37121, RRID:AB_2556549; #A11008, RRID:AB_143165; #A11037, RRID:AB_2534095; 1/1000). Nuclei were stained with Hoechst stain 33342 (Sigma), samples were mounted with Fluoromount-G (Southern Biotech), and analyzed using a confocal microscope (LSM 710, Zeiss) on an inverted stand (Axiovert 200 M, Zeiss) using objective Plan-NEOFLUAR ×20/×40 1.3 oil DIC. Images were acquired using Zeiss software ZEN 2009 and processed using AxioVision software (Zeiss). Image analyses were performed using ImageJ^31^; (RRID: SCR_003070).

### **RNA Fluorescence in situ hybridization (FISH)**

For quantitative high-content screening microscopy analyses cells were fixed for 10 min with 1% Formaldehyde (Carl Roth Inc.) at room temperature. After washing twice with PBS cells were permeabilized with 70% ethanol at 4 °C overnight. For subsequent RNA smFISH stainings, cells were washed with Stellaris Wash buffer A (BioSearch Technologies) for 5 min at room temperature. Stellaris Hybridization Buffer (BioSearch Technologies) containing the RNA binding probe (1:500) was added and the cells were incubated at 37 °C (without CO_2_) overnight. After an additional washing with Stellaris Wash buffer A, nuclei were stained with Hoechst stain 33342 (1:1000 in Stellaris Wash buffer A) for 5 min. The cells were washed with Stellaris Wash Buffer B for 5 min at room temperature. Plates were stored in PBS at 4 °C until microscopy analyses. Sequences of the SatIII RNA binding probe are provided in Supplementary Table 1.

### **FISH on formal fixed and paraffin embedded (FFPE) tissues**

**Fig. 1:**
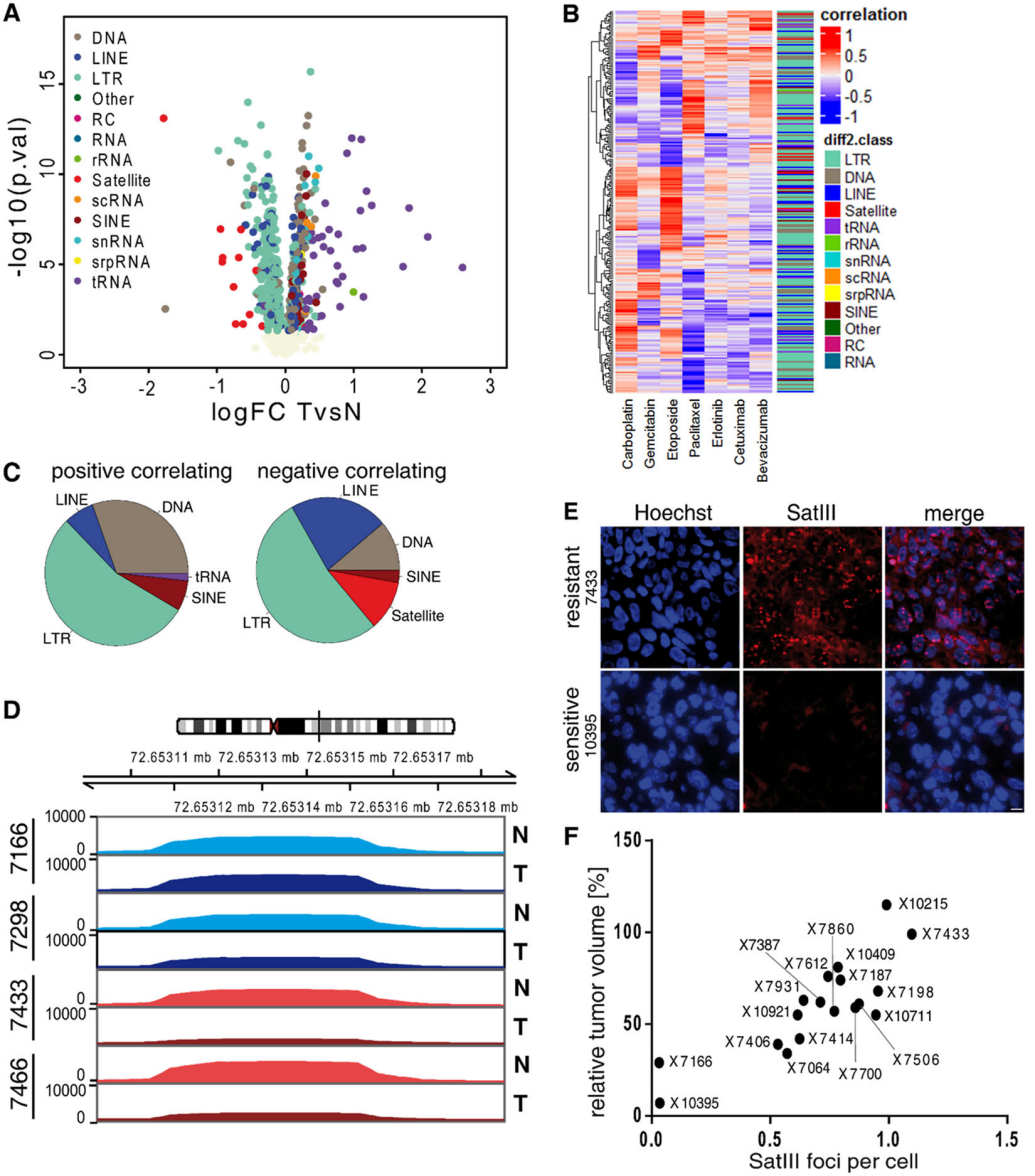
Hypomethylation of pericentromeric satellite repeats correlates with etoposide resistance in non-small cell lung cancer PDX mouse models. **A** Volcano plot shows the global methylation changes at repetitive elements between 22 patient-derived NSCLC xenograft tumor samples and their corresponding normal tissue (TvsN). Each dot represents one repetitive element, based on the RepeatMasker database, classified into subclasses (color code). The log fold change of methylation in tumor versus normal tissue is plotted on the x-axis, the y-axis shows the negative log 10 of the *p*-value. **B** Heatmap shows the Pearson’s correlation between the sensitivity of the PDXs (measured by the differential methylation between tumor and normal tissues) with the chemotherapeutics indicated by the column name. The adjacent bar indicates the repetitive region class. **C** Composition of the significantly correlated (*p*-value < 0.05) repeats classes: positive (left) and negative (right) correlations between response to Etoposide and differential methylation. **D** Methylation levels of two etoposide sensitive PDXs (7166 and 7298; green) and two etoposide resistant PDX models (7433 and 7466; red) at the Satellite III DNA locus on chromosome 9. Lighter colors represent the coverage of normal tissue (N), darker colors show the coverage of tumor samples (T). **E** RNA FISH staining of Satellite III transcripts (SatIII RNA) in FFPE tumor material of untreated etoposide resistant (7433) and etoposide sensitive (10395) PDX mice. The tissue was stained with SatIII RNA FISH probes (red) as well as Hoechst stain 33342 (blue). Scale bar, 10 µm. **F** Correlation of SatIII RNA foci and the relative tumor volume on native FFPE PDX tumor tissue. SatIII RNA foci were stained with SatIII RNA FISH, quantified, and set into relation to the response rate towards etoposide.

Each of the PDX tissues analyzed with FISH directly corresponds to the samples analyzed in the former sections (Fig. 1) and originates from Grasse et al. 2018^22^. FFPE tissues were cut with a microtome in order to acquire tissue slides with a thickness of 3 µm and mounted onto a microscope slide. Deparaffinization was performed by immersing the slide-mounted-tissue in 100% Xylene for 10 min. The slide was first immersed in 100% EtOH twice (10 min), then in 95% EtOH (10 min), and then in 70% EtOH for 1 h to permeabilize the tissue section. After a washing step with PBS (5 min), the slide was immersed in pre-warmed proteinase K solution (10 µg/mL, Enzo) and incubated at 37 °C for 20 min, followed by another washing step with PBS. Hybridization was performed as described above. Analyses were performed using a confocal microscope (LSM 710, Zeiss) on an inverted stand (Axiovert 200 M, Zeiss) using objective Plan-NEOFLUAR ×20/×40 1.3 oil DIC. Images were acquired using Zeiss software ZEN 2009 and processed using AxioVision software (Zeiss).

### Quantitative high-content screening microscopy

Plates were scanned using a Thermo Fisher Cellomics ArrayScan XTI with LED light source and 2 × 2 binned images of 1104 × 1104 pixels were acquired with a ×20 objective (Zeiss) and analyzed using the Cellomics software package (Colocalization V.4 or SpotDetector V4.1 Bioapplication). Cell nuclei were identified by Hoechst staining and according to the object identification parameters size: 100–1200 μm^2^, ratio of perimeter squared to 4π area: 1–3, length-to-width ratio: 1–3, average intensity: 500–10,000, total intensity: 3 × 10^5^–3 × 10^7^. Nuclear stress bodies were identified within a circular region extending the nucleus by maximally 20 μm. The object identification parameters for nSB were: 0.5–20 μm^2^, the ratio of perimeter squared to 4π area: 1–3, length-to-width ratio: 1–3, average intensity: 500–15000, total intensity: 5 × 10^2^–5 × 10^4^.

### Quantitative RT-PCR

The dsDNA-binding dye SYBR Green, which is included in the GoTaq qPCR Master Mix (Promega), was used to detect accumulating PCR fragments. Five ng of reverse transcription cDNA were mixed with 300 nM primers and ×1 GoTaq qPCR Master Mix to a final reaction volume of 10 µl. Cycling parameters were used according to the manufacturer’s protocol. Sequences of qPCR primers are provided in Supplementary Table 1.

### **Pyrosequencing**

Bisulfite conversion and pyromark PCR were performed with the EpiTect® Bisulfite Kit (QIAGEN) and the Pyromark PCR kit (QIAGEN) according to the manufacturer’s recommendations. The Pyromark PCR product was bound to sepharose beads on a 96-well plate utilizing the biotinylation of either forward or reverse primers. The bound PCR products were shaken on a plate shaker for five minutes at room temperature. In the meantime, 10 μM of sequencing primers were diluted in annealing buffer and added onto a separate PSQ 96-well plate (Biotage). A vacuum filter station was used for washing off the PCR product bound to the sepharose beads. The filters were washed with water and then immersed into the DNA solution on the first 96-well plate. The filters were immersed into 70% ethanol, 0.2 M NaOH, and washing buffer containing 10 mM tris-acetate (pH 7.6). The vacuum was turned off and the filters were placed into the PSQ 96-well plate containing the diluted sequencing primers. The plates were then placed on a heating block for 2 min at 85 °C. The pyrosequencing reaction was performed in a PSQ HS 96ATwo Pyrosequencer and analyzed using PSQ HS 96A software (Biotage). The sequence of the Pyromark PCR and pyrosequencing primer are provided in Supplementary Table 1.

### Statistics

Statistical analyses were performed using the GraphPad Prism 7 software package (GraphPad Software, RRID:SCR_002798). The type of statistical analyses, parameters, and number of replicates are indicated in the figure legends. For all tests, *p* value significance was defined as follows: not significant (n.s.) *p* > 0.05; *p < 0.05; **p < 0.01; ***p < 0.001.

## Results

### Hypomethylation of pericentromeric satellite repeats correlates with etoposide sensitivity

The hypomethylation of repetitive DNA is a frequent and early event in tumor development, likely affecting proliferation rates, therapy resistance, and early metastasis events. Because some lung tumors present with an intrinsic therapy resistance without prior exposure to chemotherapies, we hypothesized that the methylation of repetitive DNA is altered in cancer. Using therapy-response data for seven chemotherapies from 22 NSCLC-PDXs with available genome-wide DNA methylation data derived through methylated DNA immunoprecipitation and sequencing (MeDIP-Seq) of tumor and patient’s corresponding normal tissue^22^, we focused on the repetitive regions of the genome and calculated the differential methylation of 1116 repeating elements classified into 13 repeat classes by using the RepEnrich2 tool^24^. Global analysis revealed 690 significantly differentially methylated repetitive regions in NSCLC-PDXs compared to the matching normal lung biopsies (Fig. 1A). The strongest hypomethylation was observed at satellite DNA repeats followed by long terminal repeats (LTR), whereas the strongest hypermethylation was found in DNA regions encoding tRNAs. Though hypomethylation of long interspersed nuclear elements (LINEs), in particular of LINE-1, is a common characteristic of human cancers; we did not observe hypomethylation of LINEs (Fig. S1A). By looking at the methylation patterns of LINE-1 subclasses, we discovered that the primate-specific LINEs showed a pronounced and significant hypomethylation as previously shown in cancer^32^. The responsiveness to etoposide, paclitaxel, cetuximab, gemcitabine, carboplatin, and bevacizumab was examined by measuring the relative tumor volume as described by Grasse et al.^22^. Using the dose–response data of the 22 NSCLC-PDXs along with the differential methylation pattern of tumor tissues, we calculated the correlation between the relative tumor volume after treatment with these chemotherapeutics and the corresponding methylation values for each PDX. We found 265 significantly correlating repeats for at least one chemotherapeutic drug (Fig. 1B, Fig. S1B). The most significantly correlating repeats were detected for etoposide (95 repeats), in which LTR, LINE, and satellite DNAs were differentially methylated^32^. Interestingly, a hypomethylation of the *SatIII* locus correlated with an etoposide resistant phenotype. This was reflected by a negative Pearson correlation coefficient (Fig. 1C). The transcription of pericentromeric SatIII repeats upon HS is highly asymmetrical and most of the transcripts contain the G-rich strand of the repeat which are constantly associated with nSBs. Opposing to this, complementary C-rich transcripts are only modestly increased after stress^8^. Thus, subsequent bioinformatics analyses focused on the G-rich (GAATG) repeats. Along with GSATII, LSAU, and (CATTC)n repeats (Fig. S1C), we found the (GAATG)n repeats (SatIII) to be one of the most significantly negatively correlating repeats between methylation levels in tumor tissue and etoposide sensitivity (Figs. S1D, E). We calculated the methylation levels of the *SatIII* DNA locus on chromosome 9 and visualized them in two of the most resistant and two of the most sensitive PDXs (Fig. 1D). We found nearly no changes in methylation levels in etoposide sensitive PDX tumors versus the corresponding normal tissue, whereas the resistant PDX tumors showed strong hypomethylation in comparison to the normal tissue.

Based on the strong hypomethylation of the SatIII region in etoposide resistant tumors we hypothesized that there is a correlation between SatIII RNA expression and the resistance towards etoposide. Thus, we used single molecule RNA FISH against SatIII RNA and found a high number of SatIII foci in tissues originating from etoposide-resistant PDX tumors (Fig. 1E, F). Vice versa, a low number of SatIII foci was observed in etoposide-sensitive PDX tumors. Furthermore, using the publicly available RNA expression dataset from Klijn et al.^21^, we analyzed the relationship between SatIII RNA expression and the sensitivity to etoposide in different non-metastatic NSCLC cell lines. We used the IC50 sensitivity data from the Cancerrxgene database for erlotinib, gemcitabine, etoposide, cetuximab, paclitaxel, and cisplatin to correlate the expression with chemosensitivity towards specific chemotherapeutic drugs. Similar to what we observed for our PDX models, we found a positive correlation between etoposide response and SatIII RNA expression in the tested NSCLC cancer cell lines (data not shown), further supporting a role of SatIII expression in etoposide resistance.

### Hypermethylation of the *SatIII* DNA locus diminishes SatIII RNA expression and increases etoposide sensitivity

**Fig. 2:**
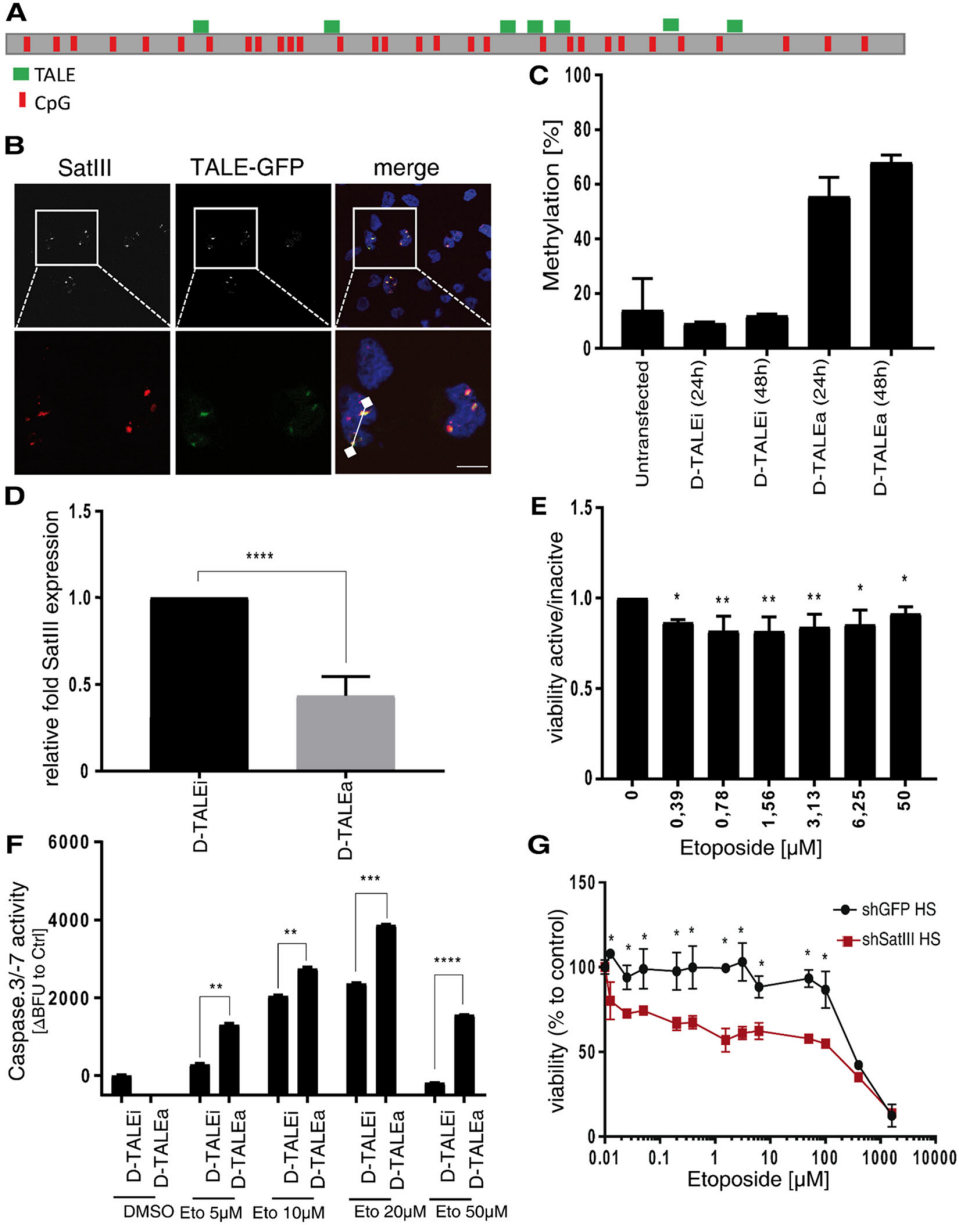
Hypermethylation of the *SatIII* DNA locus by D-TALES diminishes SatIII RNA expression and increases sensitivity towards etoposide. **A** Schematic representation of *SatIII* DNA locus with all CpG localizations (red) as well as the TALE-binding sites (green). **B** Representative images of the co-localization of TALE-GFP (green) and SatIII RNA (red). HeLa cells were transfected with the TALE-GFP construct and exposed to HS conditions (1 h at 44 °C) 24 h after transfection in order to induce SatIII RNA expression and foci accumulation. Cells were fixed, immunostained, and imaged. The histogram indicates co-localizations, represented by overlapping peaks of fluorescence intensities. **C** Methylation level of the *SatIII* DNA locus in D-TALEa (active) and D-TALEi (inactive vector control) transfected U2OS cells. At 24 h and 48 h post-transfection U2OS cells were exposed to 44 °C for 1 h (HS) conditions and harvested. Cells were FACS-sorted and mCherry positive cells used for DNA extraction and subsequent pyrosequencing of the SatIII DNA locus. Shown is the percent of methylation to a transfection control. **D** Quantitative PCR analyses for SatIII RNA expression of samples from (**C**). Samples incubated for 48 h with the transfection mix were used. Error bars represent standard deviation of the mean of two individual replicates. **E** Drug response of U2OS cells either transfected with D-TALEa or an inactive control plasmid (D-TALEi). Viabilities were examined by AlamarBlue. Error bars represent standard deviation of the mean of three replicates. Two-tailed paired Student’s t test significant *P*-values are marked: < 0.05 with (*), < 0.01 with (**), < 0.001 with (***). **F** Caspase-3/-7 assay of U2OS cells either transfected with the D-TALEa plasmid or an inactive control (D-TALEi). At 24 h post-transfection, cells were treated with different concentrations of etoposide or a DMSO control for 24 h. Depicted are the differences of blue fluorescence (BFU) signals between D-TALEa and D-TALEi. Error bars represent standard deviation of the mean of the three replicates. Two-tailed paired Student’s *t*-test significant *P*-values are marked: < 0.05 with (*), < 0.01 with (**), < 0.001 with (***). **G** Cell viability of HCC827 cells stably expressing either shRNA which targets SatIII (shSatIII, blue) or a shGFP-Control (red). Cells were exposed to 44 °C for 1 h (HS) and afterwards immediately treated with the indicated etoposide concentrations. After an additional 48 h cell viability was measured using AlamarBlue.

We reasoned that a reversion of the hypomethylation should decrease SatIII expression and alleviate etoposide resistance. To address this, we used transcription-activator-like effectors (TALEs), DNA binding domains originally derived from *Xanthomonas* bacteria which contain a modular domain of repeats that each recognize one nucleobase via a repeat variable di-residue (RVD), and can be programmed to bind user-defined DNA sequences^33^. We designed TALEs fused to a DNMT3a3L (D-TALE) construct for specific binding to the *SatIII* DNA locus (Fig. 2A, Fig. S2A)^34^. This enabled targeted methylation of the *SatIII* DNA locus. Immunostaining was used to verify the correct localization of the TALE at the desired locus^35,36^. HeLa cells, which are the main model system used for studies on nSBs and the heat stress response^37^, were transfected with the TALE construct fused to a GFP-tag and subjected to HS conditions (1 h at 44 °C). These conditions were chosen based on preliminary experiments and previous results by Hussong et al. that had determined these conditions to be sufficient to strongly induce SatIII expression and nSB formation^13^. In parallel, cells were stained with a SatIII smFISH. Microscopy analyses revealed co-localization of the TALE constructs and the SatIII RNA FISH foci (Fig. 2B; Fig. S2B). To confirm our hypothesis that artificial methylation of the *SatIII* locus results in a decreased *SatIII* expression and an increased etoposide sensitivity, we screened cell lines for their baseline methylation level. Whereas HeLa cells were found to carry 18% methylation at the targeted SatIII locus, U2OS cells and HEK293T cells, which are used as an alternate HS model, had approximately 5% methylation at the *SatIII* locus, providing increased capacities for artificial methylation (Fig. S2C). Therefore, we chose to use U2OS and HEK293T cells to intensify the methylation capacity of the D-TALEs. To exclude toxic effects originating from variable transfection efficiencies, we performed cytometer assays, acquiring information on the percentage of transfected cells and determining the viability of these cells with DAPI staining of the cells prior to the measurement. Transfection efficiencies were constantly around 80%, and in the same range was the percentage of living cells on the whole population. This applied for both control (DNMT inactive) and DNMT- active settings (Fig. S2E, F). The methylation level of the *SatIII* locus increased 4-fold 24 h after transfection with the active D-TALE (D-TALEa) construct and 5-fold after 48 h, compared to an identical TALE construct bearing a E756A mutation in the DNMT3a domain that renders this construct catalytically inactive (Fig. 2C)^38^. The methylation levels at LINE1 elements were used as control for methylation specificity. Since we were unable to detect any change in cytosine methylation in cells transfected with either the active or the inactive D-TALE, we confirmed a locus-specific hypermethylation of the *SatIII* locus (Fig. S2D). Along with increased locus methylation, D-TALE transfected cells showed a decreased expression of SatIII compared to the inactive control plasmid (Fig. 2D). Further on, we compared the drug response of U2OS cells transfected either with the active or the inactive D-TALE plasmid. Viabilities of the former cells displayed a moderate and significantly enhanced sensitivity towards etoposide compared to the inactive control (Fig. 2E). Etoposide treatment causes DSBs and ultimately triggers apoptotic pathways which are either TP53 dependent or independent^39^. Cells transfected with D-TALEa displayed an enhanced Caspase-3/-7 activity after etoposide treatment compared to the inactive control, which reflects an increased apoptotic activity in the cells transfected with the D-TALEa (Fig. 2F).

### Knockdown **of SatIII RNA sensitizes cells towards etoposide**

To gather further evidence that not only methylation but the actually altered expression of SatIII (and subsequent changes in the recruitment to nSBs) affected the responsiveness to etoposide, we transfected post-HS HeLa cells with either a siRNA targeting *SatIII* RNA transcript or a non-targeting control siRNA. Along with the decreased expression and foci formation of SatIII RNA after SatIII knockdown in response to etoposide treatment, the siRNA SatIII-treated cells showed significantly reduced cell viability (Fig. S3A, C, D, E). In order to validate these findings, we performed a knockdown of SatIII using antisense-oligos targeting SatIII (ASO-SatIII) (Fig. S4A–C). Transfection with ASO-SatIII led to a higher sensitivity towards etoposide compared to a non-targeting control, which was observed both in an AlamarBlue cell viability assay and a microscopy-based proliferation assay (Figs. S4D-F). An even stronger effect of SatIII expression on etoposide treatment response was observed in NSCLC HCC827 cells upon a stable shRNA mediated SatIII repression (Figs. 2G and S5C). HCC827 cells were selected as a model serving as an etoposide resistant cell line (Fig. S5A), illustrated by a considerably higher IC50 compared to H2030, an etoposide sensitive NSCLC cell line (Fig. S5B). In contrast to the effects observed for SatIII repression, transient overexpression of the SatIII transcripts resulted in increased cell viability compared to the control (Fig. S3B, D). These effects were generally intensified at lower etoposide concentrations, strongly suggesting that cell death effects at higher concentrations overrule SatIII functions.

### SatIII recruits TOP2A to nSB foci and protects cells from DNA double strand breaks

**Fig. 3:**
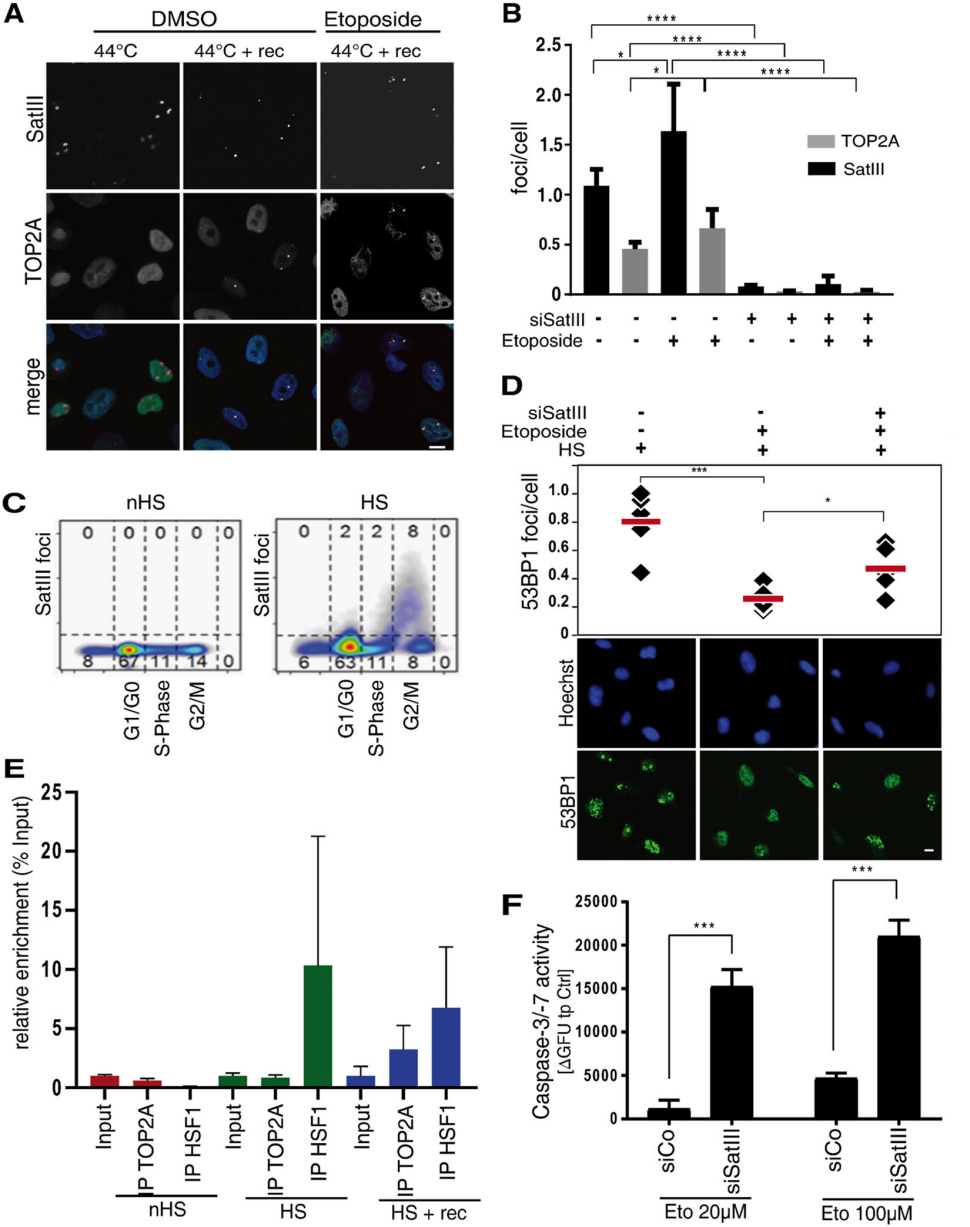
SatIII recruits TOP2A to nSB foci thereby protecting cells from DNA double strand breaks. **A** Representative images of the SatIII RNA and TOP2A co-localization in HeLa cells exposed to HS conditions (1 h at 44 °C) or HS plus 24 h recovery at 37 °C and DMSO or etoposide 10 µM treatment. SatIII RNA was stained using smFISH (red), TOP2A was stained using a protein-specific antibody (green). Scale bar, 10 µm. **B** Quantification of (**A**) by counting the number of foci per cell. Quantification was performed using an automated ImageJ pipeline, *n* = 5. **C** Cell cycle assay utilizing Hoechst staining was performed to clarify SatIII expression patterns during the cell cycle. HeLa cells were subjected to HS (1 h at 44 °C) or control conditions, fixed and stained with Hoechst staining dye. The cells were monitored over the course of the cell cycle in an automated HCS microscope. A minimum of 3000 cells, separated in *n* = 6 replicates, were quantified for each condition. **D** Effects of SatIII RNA knockdown on DNA damage was investigated by immunofluorescence staining for 53BP1. HeLa cells were transfected with siSatIII and scramble RNA (control), respectively. Cells were then exposed to HS conditions (1 h at 44 °C) or constant 37 °C and treated with 20 µM etoposide or DMSO. After 24 h, cells were fixed and stained with a protein-specific 53BP1antibody (green). Counterstaining of nuclei was performed with Hoechst stain. Imaging and analyses were performed utilizing HCS microscope-based quantifications of the staining. Error bars represent the standard deviation of the mean of three replicates. Two-tailed paired Student’s *t* test significant *P*-values are marked: < 0.05 with (*), < 0.01 with (**), < 0.001 with (***). Scale bar, 10 µm. **E** RNA immunoprecipitation in HeLa cells subjected to three different treatment conditions: HS (1 h at 44 °C), HS with a 24 h recovery time at 37 °C (HS + rec), and non-HS conditions (nHS, 37 °C). Chromatin was sheared by sonication and precipitated using an antibody against human TOP2A or HSF1. Binding to SatIII was analyzed using qPCR. HSF1 was used as a positive control. Figure shows a typical result for two biological replicates, each with three technical replicates. **F** Caspase-3/-7 assay of HeLa cells either transfected with the siRNA targeting SatIII (siSatIII) or control siRNA (siCo). After transfection, cells were treated as indicated with etoposide or a DMSO control. Error bars represent standard deviation of the mean of three replicates. Two-tailed paired Student’s t-test significant *P*-values are marked: < 0.05 with (*), < 0.01 with (**), < 0.001 with (***).

Etoposide forms a ternary complex with DNA and TOP2A during DNA replication. This prevents re-ligation of the DNA strands, leading to DSB and the induction of the DNA damage response^40^. TOP2A consistently reaches peak expression during the G_2_/M phase of the cell cycle, associates with replication forks, and remains tightly bound to chromosomes during mitosis. Experiments in *Drosophila melanogaster* have revealed that *SatIII* repeats harbor several TOP2A cleavage sites^41^ and that the displacement of HP1 and TOP2A with synthetic polyamides from the *SatIII* DNA locus results in chromatin opening and de-silencing of the nearby transcription sites^42^. Thus, to investigate whether TOP2A is changing its cellular localization we performed immunofluorescence experiments in HeLa and U2OS cells under HS conditions (1 h at 44 °C). Cells were fixed either immediately after 1 h at 44 °C or after 24 h recovery at 37 °C and subsequently stained with SatIII smFISH probes and a TOP2A antibody. Though the co-localization of TOP2A and SatIII RNA was not observed immediately after HS, recovery at 37 °C for 24 h led to a co-localization of both components in nearly 50% of all SatIII foci containing cells, with an even increased number of foci under etoposide treatment (Fig. 3A, Fig. S6A). This accumulation of TOP2A at sites of SatIII foci was dependent on SatIII expression, as a knockdown of SatIII abolished all SatIII foci and all TOP2A accumulation sites (Fig. 3B). Interestingly, western blot experiments revealed no effect of siRNA mediated SatIII knockdown on TOP2A expression (Fig. S6B). Similar to TOP2A expression patterns^43,44^, SatIII foci induction was mainly induced during late S-, and peaked in G2/M phase (Fig. 3C). The expressions of SatIII and TOP2A, therefore, occur in the same phase of the cell cycle.

In order to study the downstream effects of varying SatIII expression levels on the complex formation of DNA, TOP2A, and etoposide; we stained the DNA damage reporter protein p53 binding protein 1 (53BP1). HeLa cells were transfected with either siRNAs targeting SatIII or a non-targeting control. HS conditions (1 h at 44 °C) were applied before treatment with etoposide or DMSO as control. We found that 53BP1 co-localized with SatIII (Fig. S7A) which was also observed for gamma-H2AX as an additional DNA damage reporter (Fig. S7B). Staining quantification uncovered increased DNA damage when SatIII RNA expression was decreased (Fig. 3D; Fig. S7C). This was also true for HCC827 cells stably expressing shSatIII RNA (Fig. S7D). On the other hand, a transient overexpression of SatIII showed a significant decrease in 53BP1 foci per cell (Fig. S7E).

To further understand the interplay between SatIII and TOP2A, we performed an RNA immunoprecipitation experiment. HeLa cells were subjected to three different treatment conditions: HS (1 h, 44 °C), HS with a 24 h recovery time at 37 °C, and non-HS conditions (constant 37 °C). RNA was precipitated using antibodies against TOP2A and HSF1. Non-heat stressed cells did not show any binding between SatIII and HSF1 or TOP2A, whereas heat-stressed cells showed an enrichment of SatIII at HSF1 and TOP2A after recovery (Fig. 3E). As a downstream effect we found that knockdown of SatIII caused a significant increase in caspase-3/-7 activity upon etoposide treatment (Fig. 3F).

### BET inhibitors re-sensitize etoposide-resistant cells through SatIII down-regulation

**Fig. 4:**
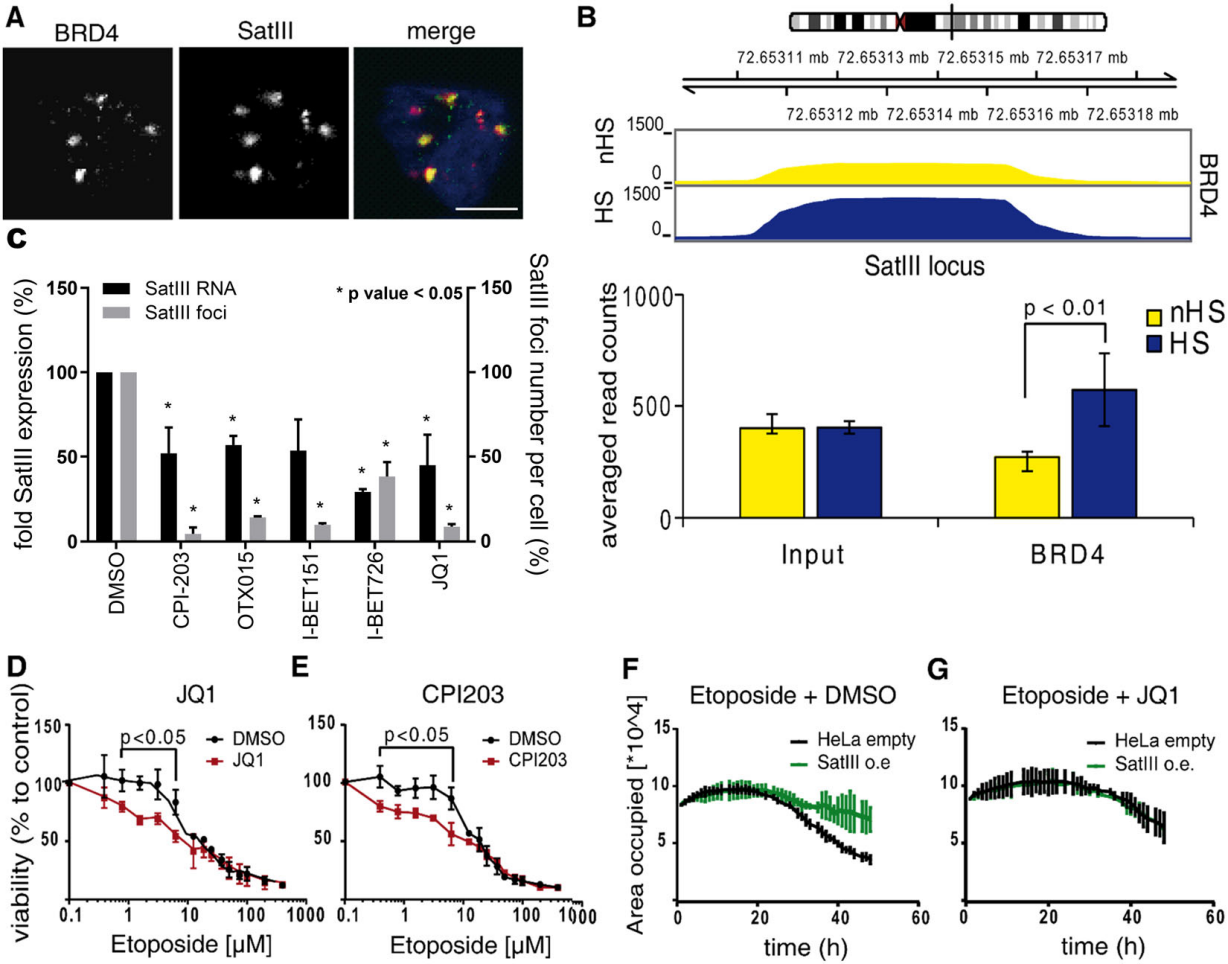
BET protein inhibitors revert etoposide resistance through SatIII regulation. **A** Representative images of co-localization of SatIII RNA and BRD4 after exposure to HS conditions (1 h at 44 °C). Immediately following HS, cells were fixed, immunostained, and imaged. SatIII was stained with smFISH (red), BRD4 with a protein-specific antibody (green). Scale bar, 10 µm. **B** Binding of BRD4 to the *SatIII* DNA locus after HS (1 h at 44 °C). For the ChIP-Seq experiment cells were subjected to HS or control conditions. ChIP was performed with a BRD4-specific antibody. **C** Effect of BRD4 inhibition on SatIII expression. HeLa cells were treated with various BRD4 inhibitors and exposed to HS conditions (1 h at 44 °C). Two read-out methods were applied: RNA expression was measured by qPCR and RNA FISH was used to quantify SatIII RNA foci. Values of non-treated cells (DMSO control) were set to 100%. Error bars represent SD of the mean of *n* = 2 independent replicates. *P*-values < 0.05 marked with (*). Significance was determined using two-tailed paired Student’s t-test. **D** Cell viability of HeLa cells treated with etoposide and the BRD4 inhibitor JQ1 (1 µM) or DMSO as control. Directly after exposure to HS (1 h at 44 °C), cells were treated with the indicated etoposide concentrations in combination with JQ1. After an additional 48 h, cell viabilities were measured using AlamarBlue. Error bars represent SD of the mean of *n* = 2 replicates. *P*-values <0.05 are marked with (*), Significance was determined using two-tailed unpaired Student’s t-test. **E** Same experiment as in (D) but with the BRD4 inhibitor CPI203 (1 µM). **F** Cell proliferation of HeLa cells either stably overexpressing SatIII RNA or an empty vector control. Cells were treated with 20 µM etoposide and cell proliferation was measured by acquisition of images every 30 min over a time course of 48 h. Confluency was analyzed with the cell profiler software. **G** Cell proliferation assay performed as described in (**F**) but with a combination treatment of 20 µM etoposide and 5 µM JQ1.

Next, we searched for a compound to reduce SatIII RNA expression and revert etoposide resistance. We tested if disrupting BRD4 binding to chromatin with small molecules results in an increase in etoposide sensitivity. BRD4 co-localized with SatIII RNA at nSBs (Fig. 4A) and displayed a specific increased binding to the *SatIII* DNA locus at chromosome 9 with ChIP-Seq analysis (Fig. 4B). Furthermore, a significant decrease of SatIII RNA expression under BRD4 inhibition was revealed by qPCR and quantitative high-content microscopy showed a significant reduction of SatIII foci numbers (Fig. 4C, Fig. S8A). To examine whether the effect of BRD4 inhibition on SatIII expression also impacts the response towards etoposide, we performed cell viability assays and added the BRD4 inhibitor JQ1 in combination with etoposide. Similar to siRNA silencing, the inhibition of SatIII expression by JQ1 caused a higher sensitivity towards etoposide compared to the DMSO control (Fig. 4D). The same effect was observed for treatment with CPI203, another small molecule inhibitor of BRD4 (Fig. 4E). Vice versa, stable overexpression of SatIII resulted in substantially better cell survival under etoposide treatment (Fig. S8B). In this assay, cell proliferation was traced by means of confluency (area occupied) measurements. Visual inspection showed neither increase in cell size nor a major impact on cell morphology. This confirmed our previous observations of improved cell proliferation of *SatIII* overexpressing cells compared to an empty control upon etoposide treatment (Fig. 4F). This effect was reverted when we applied a combination treatment of etoposide and the BRD4 inhibitor JQ1 or CPI203. (Fig. 4G, Fig. S8C-E). We concluded that BRD4 inhibition re-sensitizes cells for etoposide treatment. Further validation was provided in a proliferation assay in NSCLC lung cancer cell lines where HCC827 was the etoposide resistant and H2030 the etoposide sensitive cell line. Knocking down SatIII in HCC827 cells led to a decreased survival under etoposide treatment in comparison to a shGFP control. The opposite effect was observed for H2030 cells stably overexpressing SatIII (Fig. S8F, G).

## Discussion

**Fig. 5:**
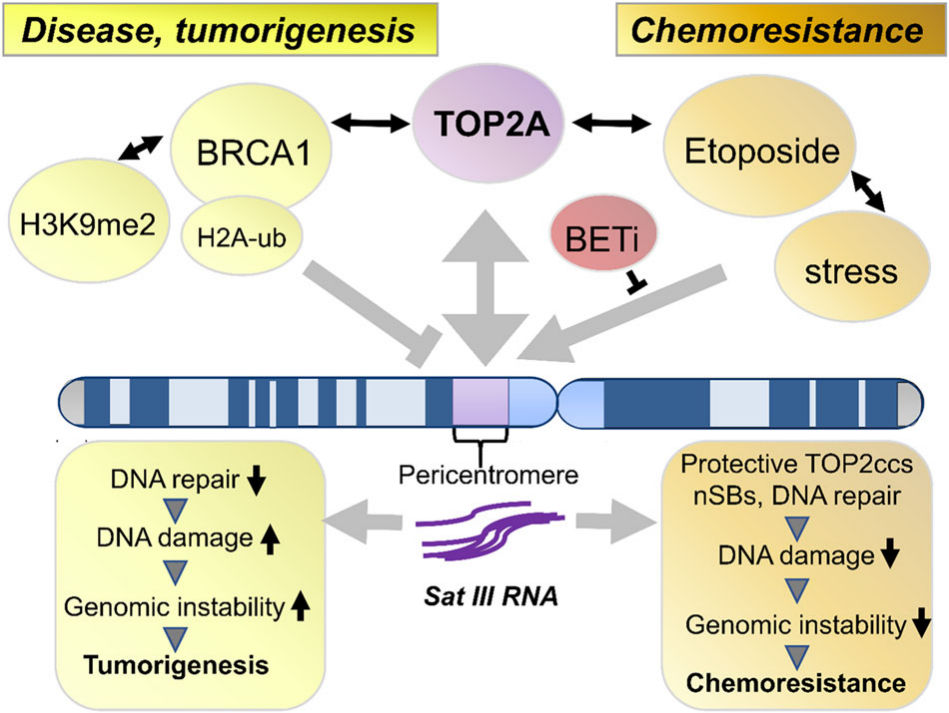
Model of SatIII regulation in tumorigenesis and chemoresistance. Loss of BRCA1 results in an increased SatIII RNA expression through reduced ubiquitination of H2A and a relaxation of pericentromeric heterochromatin, reflected by a loss of H3K9me2 (left side, Zhu et al., 2018, Padeken et al.). SatIII RNA interacts with the BRCA1-associated protein network and destabilizes replication forks which in turn enhances DNA damage and genomic instability, ultimately promoting tumor growth. Etoposide also drives SatIII expression, but in this case, SatIII RNA facilitates the recruitment of TOP2A to TOP2ccs located at nSBs (our data). This leads to less DNA damage and subsequent downstream mechanisms that decrease genomic instability and therefore cells are more resistant against etoposide.

In this study we report that SatIII RNA induces etoposide resistance in lung cancer. We demonstrate that the recruitment of TOP2A through SatIII to nSBs is associated with a reduction in 53BP1 formation, ultimately promoting decreased apoptosis activation and increased cell survival. We propose a model in which TOP2A and SatIII RNA form protective complexes that are either inaccessible to downstream DNA damage response activation or lead to a local accumulation of TOP2ccs in distinct DNA regions accompanied by an overall reduction of TOP2ccs in other DNA regions. Both models support that SatIII functions as a “sponge” by capturing TOPccs and protecting cells from the initiation of DNA damage response pathways (Fig. 5). TOP2 chromatin localization and its trapping in stable TOP2cc has been found to occur independently from transcription such that the recruitment of TOP2A to nSBs through *SatIII* seems not to be a consequence of the active transcriptional process during HS^45^. Though Satellite RNA expression has been described as increased in a broad range of cancer entities^6,14^, its functional role remains unclear. As a possible explanation for the increased SatIII expression in tumors, Zhu et al. investigated BRCA1 deficiency and found a decrease of ubiquitin-histone H2A at satellite repeats and a diminished heterochromatin structure^7^. This results in an increase in satellite expression, followed by induction of the DNA damage response and genomic instability^6,7^. Similarly, H3K9me2 loss is synergistically lethal with BRCA1 and fosters a de-repression of satellites, the formation of RNA:DNA hybrids, and genomic instability^4^. An immediate effect of dysregulated H3K9me2 on lung cancer development might be due to aberrant Satellite expression^46^. Intriguingly, a strong hypomethylation of satellite DNA is prevalent in ovarian and urothelial carcinoma^47^. As our current work shows, a *SatIII* locus-specific DNA methylation with TALEs fused to DNA methyltransferases renders cells sensitive to etoposide and impacts cell viability and apoptosis. Our finding that expression and DNA methylation are altered in PDXs prior to etoposide treatment suggests that naïve tumor cells bear an intrinsic resistance towards etoposide. This highlights SatIII as a potential biomarker for etoposide treatment success. The observed increase in SatIII RNA could be induced by oncogenic stress, but is most likely independent of BRCA1 status, because BRCA1 deficiency has been described in connection with increased chemosensitivity^48^. Previous studies suggested different mechanisms for etoposide resistance, including modified TOP2 expression, increased expression of ABC transporters, and decreased expression of genes involved in DNA mismatch repair^49–51^. By affecting TOP2 localization SatIII directly affects TOP2 functionality, and functions alongside other resistance mechanisms.

The clinical use of etoposide in cancer treatment is not restricted to lung cancer, but includes a large variety of other cancer entities, i.e. Hodgkin and non-Hodgkin lymphoma, testicular cancer, Ewing’s sarcoma, and others. A correlation of SatIII expression with therapy resistance data in these tumors will be a promising and intriguing approach in determining potential roles of satellite RNAs in therapy resistance. A combination treatment of etoposide and BRD4 inhibitors reversed the resistance phenotype in vitro, making this approach a powerful candidate for clinical application^52^. It cannot be excluded that the inhibitory effect of BRD inhibition is also partly due to a general effect on gene expression. However, several findings speak against a more global effect: We found (i) a co-localization of BRD4 and SatIII upon stress, (ii) repression of SatIII RNA expression and foci formation upon multiple other BRD4 inhibitors as well as upon a siRNA based BRD4 knockdown, and (iii) an induction of resistance upon expression of SatIII. Further work is needed to provide additional options for co-treatments or to develop specific inhibitors of SatIII RNA that overcome therapy resistance in cancer patients by intervening with heterochromatin formation at the *SatIII* locus.

## Supplementary information

Supplemental_Data

Supplemental Table1
