# Supplementary material for: Pericentromeric Satellite III transcripts induce etoposide resistance
Source: Cell Death Dis. 2021 May 24;12(6):530. doi: 10.1038/s41419-021-03810-9 (PMC8144429; doi:10.1038/s41419-021-03810-9)
Supplement: Supplementary file 1 — Supplemental_Data [file 41419_2021_3810_MOESM1_ESM.docx]

**SUPPLEMENTAL INFORMATION**

Pericentromeric Satellite III transcripts induce etoposide resistance

**Julian Kanne, Michelle Hussong, Jörg Isensee, Álvaro Muñoz-López, Jan Wolffgramm, Felix Heß, Christina Grimm, Sergey Bessonov, Lydia Meder, Jie Wang, H. Christian Reinhardt, Margarete Odenthal, Tim Hucho, Reinhard Büttner, Daniel Summerer, Michal R. Schweiger**

**

**

**Supplemental Figure 1. Related to Figure 1. SatIII Methylation and expression correlates with etoposide therapy sensitivity.**

**A** Differential methylation of LINE elements between 22 NSCLC-PDX tumor samples and their corresponding normal tissue (TvsN). On the y-axis, the log2 of methylation changes tumor versus normal are shown. The x-axis shows the different subclasses of LINE elements. CR1 = chicken repeat 1; L1M = LINE1 mammalian, L1P = Line1 primate; L2 = LINE2. **B** Heat map showing the corresponding p-values to the Pearson’s correlation values of the sensitivity of the PDXs towards the indicated chemotherapeutics and the level of differential methylation between tumor and normal tissues on repetitive elements **C** Heat map showing differential methylation of the significantly correlating satellite repeats. The bar plot demonstrates the % of tumor volume of each PDX after treatment with etoposide. **D** Correlation plot of methylation changes (TvsN) of the Satellite III repeat (GAATG)n and sensitivity (% tumor volume) of the PDXs towards etoposide. Methylation score (TvsN) is plotted on the x-axis. On the y-axis the % tumor volume after treating the PDX with etoposide. Each dot represents one PDX. The Pearson’s correlation coefficient (r) as well as the corresponding p-value (p) are indicated.**E** Correlation plot of the methylation changes (T vs N) of of the GSATII, LSAU, and (CATTC)n repeats and the sensitivity (% tumor volume) of the PDX towards etoposide. The methylation score (TvsN) is plotted on the x-axis. On the y-axis the % of tumor volume after treatment of the PDX with etoposide. Each dot represents one PDX. The Pearson’s correlation coefficients (r) and the corresponding p-values are shown.


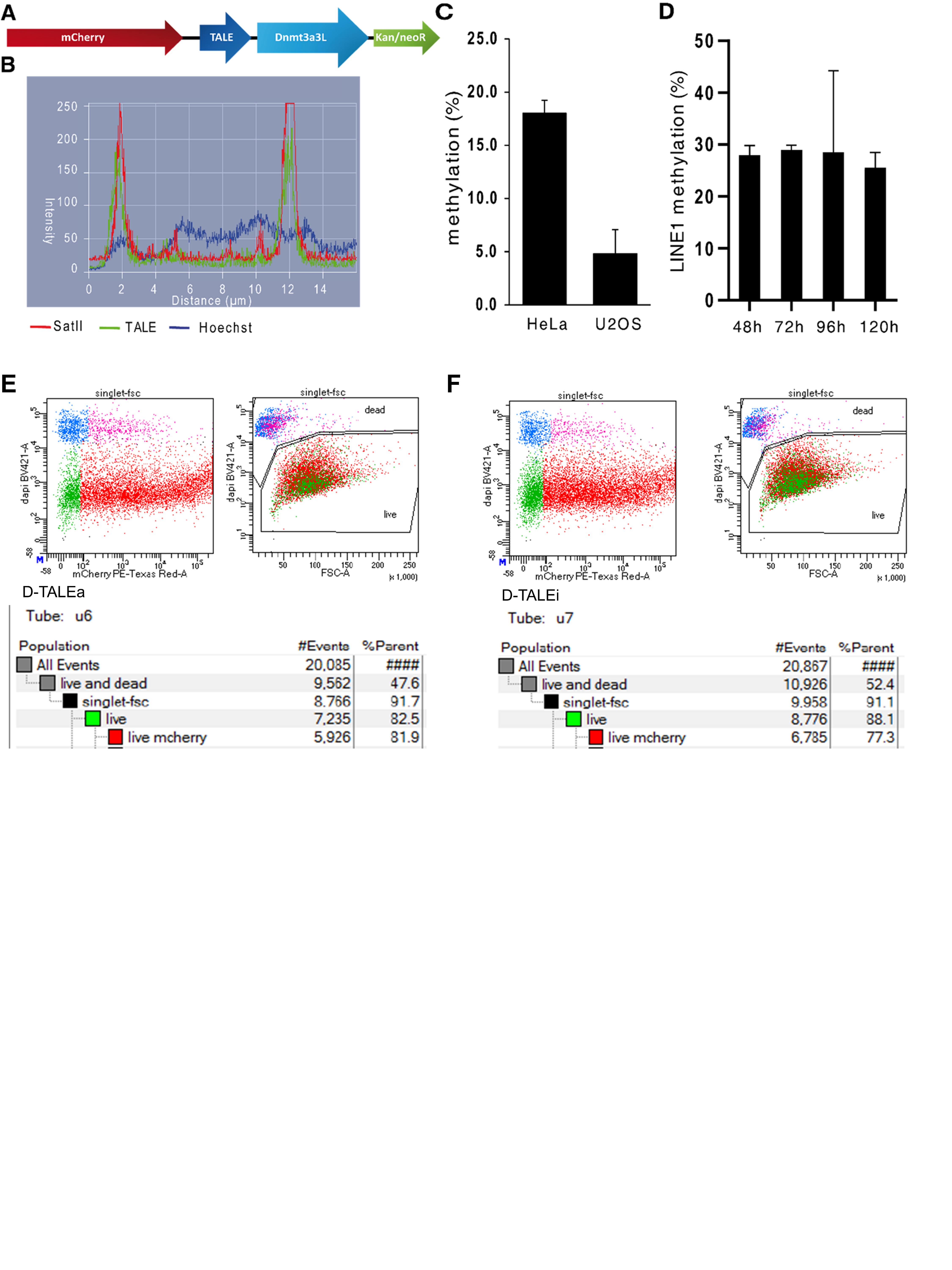


**Supplemental Figure 2. Related to Figure 2. Site-Specific DNA methylation of the SatIII locus with D-TALEs**

**A** Schematics of the D-TALE vector. **B** Histogram of co-localisation of TALE-GFP (green) and SatIII RNA (red) (corresponding to Figure 2B) representing overlapping peaks of fluorescence intensities. **C** Percent methylation at *SatIII* DNA locus in untreated HeLa and U2OS cells measured by pyrosequencing in relation to a transfection control. **D** LINE1 methylation of HeLa cells after transfection with D-TALEa. **E** Representative FACS data plots of U2OS cells transfected with D-TALE-active. Transfection efficiency was controlled through quantification of pmcherry labeled cells, which represent transfected cells. Discrimination of live/dead cells was performed by DAPI staining. **F** Representative FACS data plots of U2OS cells transfected with D-TALE-inactive.

**
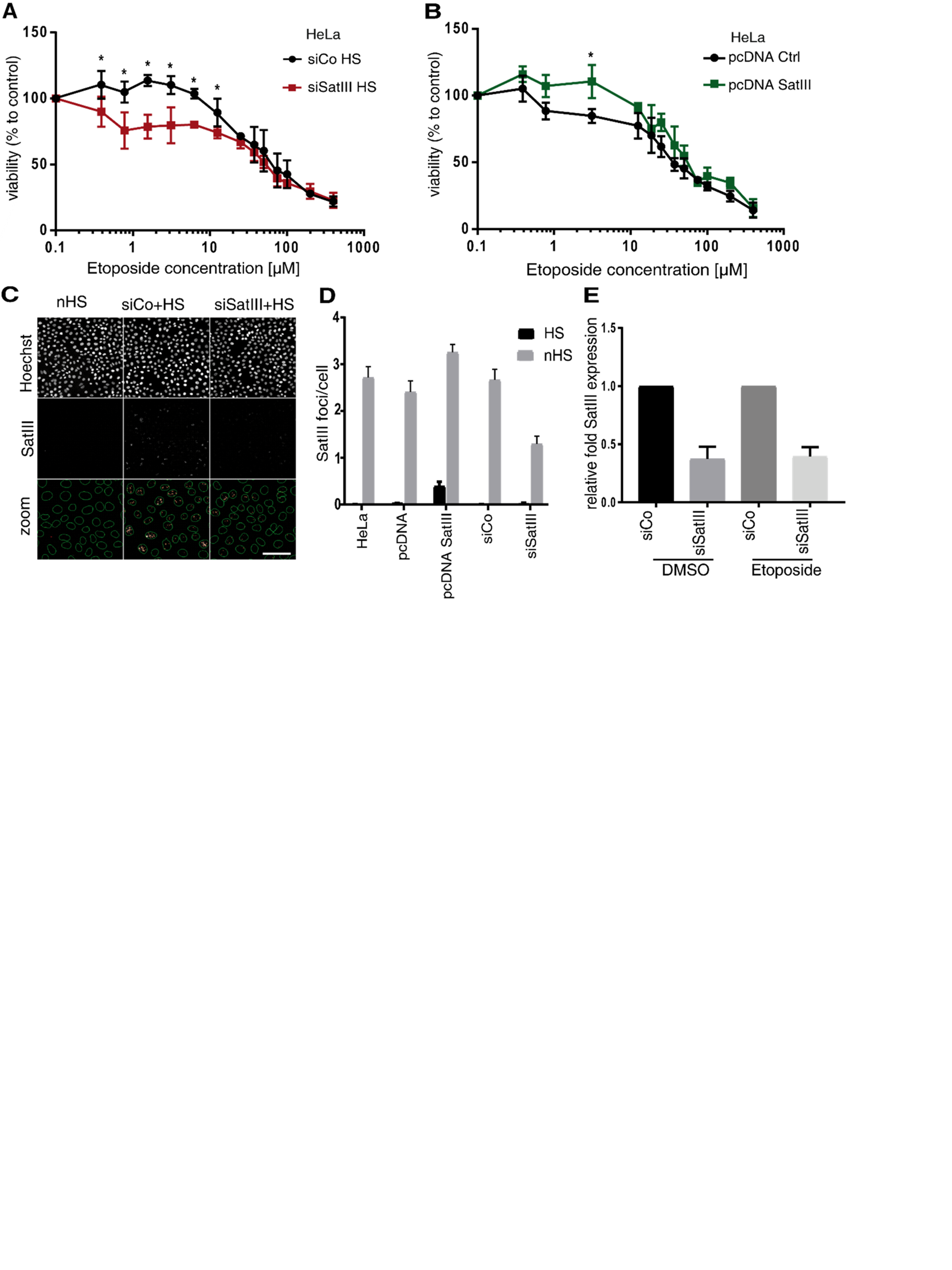
**

**Supplemental Figure 3. Related to Figure 2. Expression of SatIII RNA impacts sensitivity towards etoposide.**

**A** Cell viability of HeLa cells transfected with either siCo or siSatIII siRNAs. At 24h post-transfection cells were exposed to HS conditions (44°C for 1h) and immediately treated with the indicated etoposide concentrations. After an additional 48h cell viabilities were measured using AlamarBlue. (A,B) Values represent the mean of three independent experiments, and error bars represent the SD. P-values <0.05 are marked with (*). Significance was determined with two-tailed paired Student’s t test. **B** Cell viability of HeLa cells transfected with either an empty plasmid pcDNA (pcDNA Ctrl) or an overexpression construct pcDNA-SatIII (pcDNA SatIII) plasmid. At 24h post-transfection cells were treated with the indicated etoposide concentrations. After an additional 48h the cell viabilities were measured using AlamarBlue. **C** Representative images for the quantification of HeLa cells transfected with either siSatIII (and siCo) or pcDNA-SatIII (and empty pcDNA) under nHS and HS conditions (1h at 44°C). SatIII was immunostained by FISH. Hoechst stain was used to counterstain the nuclei. A number of SatIII foci were quantified using HCS microscope. Scale bar 50 µm. **D** Quantification of (A). Error bars represent SD of five replicates. **E** qPCR experiment of HeLa cells subjected to SatIII knockdown or control conditions. Cells were subjected to HS conditions (1h at 44°C) and RNA was isolated immediately. Shown is the relative fold SatIII expression with and without etoposide treatment. Error bars represent SD of three replicates.


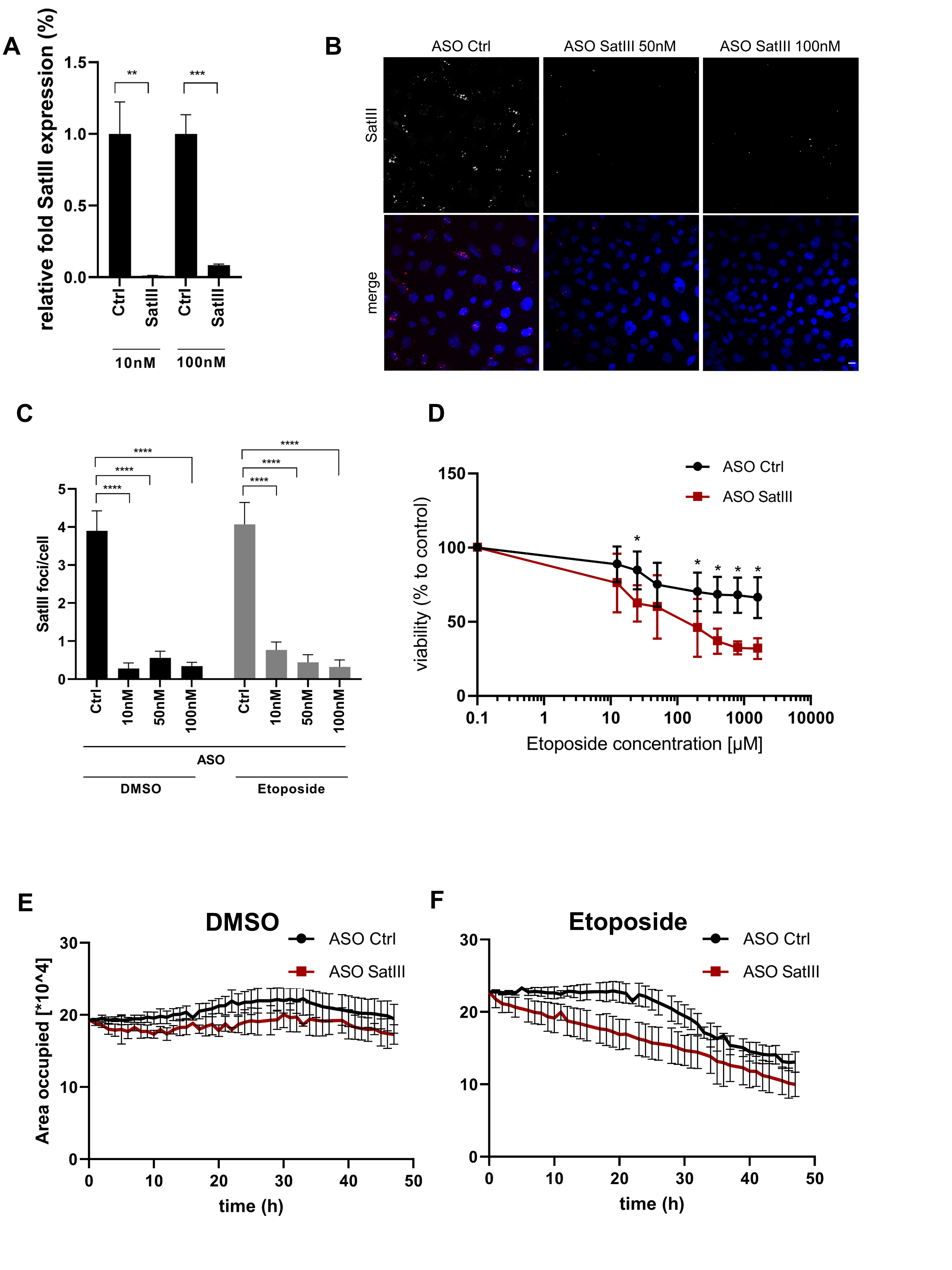


**Supplemental Figure 4. Related to Figure 2. Transfection with ASO-SatIII impacts sensitivity towards etoposide.**

**A** qPCR of heat shocked HeLa cells transfected with an antisense-oligo targeting SatIII or a non-targeting control. Different concentrations of oligos are applied (10nM and 100nM). **B** Representative images of the SatIII RNA foci in antisense-oligo SatIII/Ctrl-transfected HeLa cells exposed to HS plus 24h recovery at 37°C and DMSO treatment. SatIII RNA is stained using smFISH (red). Scale bar, 10µm. **C** Quantification of (A) by counting the number of foci per cell. Quantification is performed using an automated ImageJ pipeline, n=5. **D** Cell viability of HeLa cells transfected with either antisense-oligos targeting SatIII or a non-targeting control. Cells were exposed to 44°C for 1h (HS) and afterwards immediately treated with the indicated etoposide concentrations. After an additional 48h cell viability was measured using AlamarBlue. **E,F** Cell proliferation of HeLa cells transfected with ASO-SatIII or ASO-Ctrll and treated with DMSO or 20µM etoposide. Proliferation was measured by acquisition of images every 30 minutes over a time course of 48h. Confluency was analyzed utilizing the cell profiler software.

**
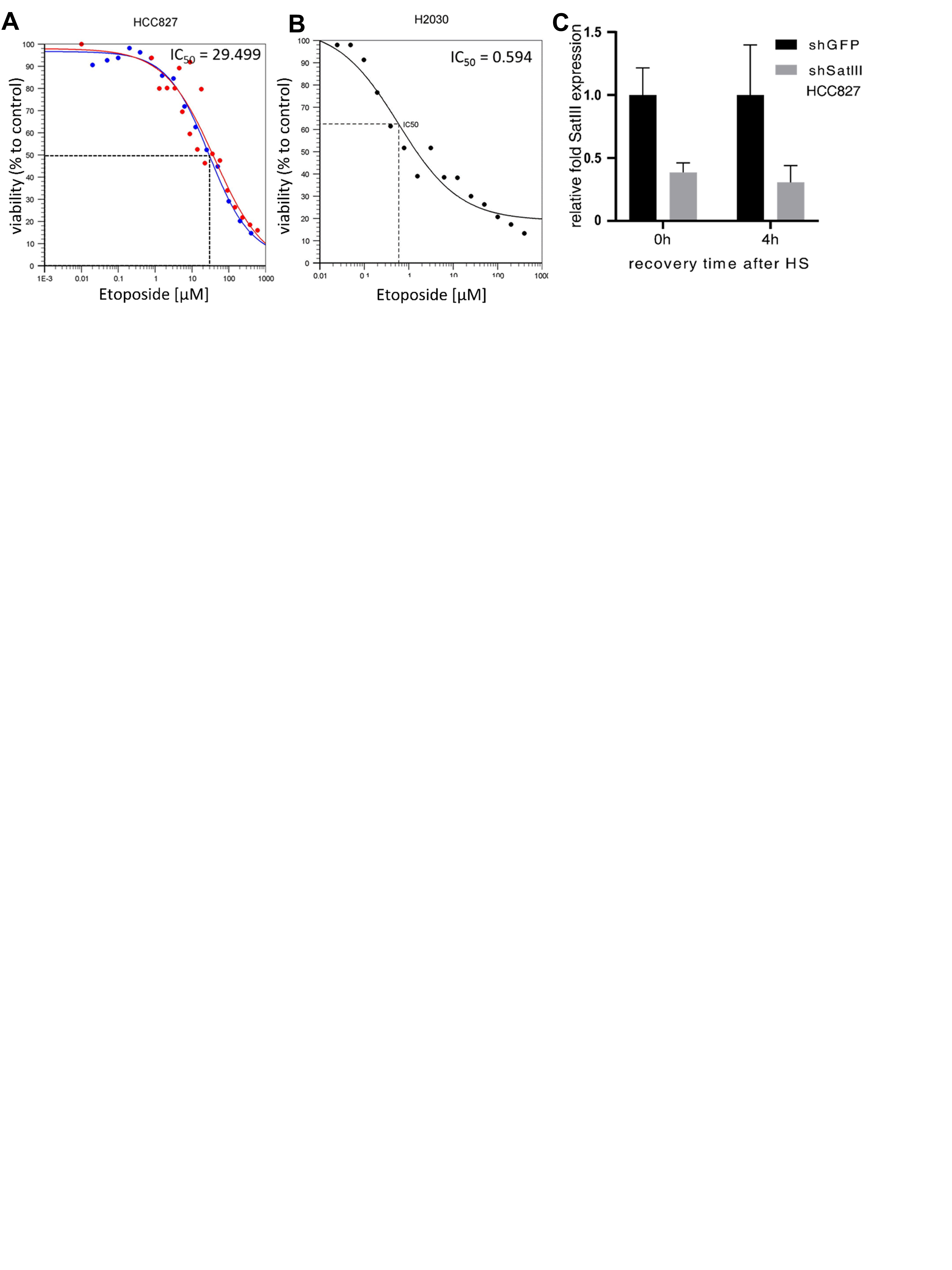
**

**Supplemental Figure 5. Related to Figure 2. NSCLC cell lines used in this study.**

**A** Dose response curves of NSCLC cell line HCC827. Cells were treated with increasing etoposide concentrations, viabilities were measured using AlamarBlue. **B** Dose response curves of NSCLC cell line H2030. Cells were treated with increasing etoposide concentrations, viabilities were measured using AlamarBlue. **C** qPCR experiment of HCC827 cells stably expressing either shRNA, which targets SatIII (shSatIII), or a shGFP-Control. Cells were subjected to HS conditions (1h at 44°C) and RNA was isolated immediately or after a 4h recovery at 37°C. The relative fold SatIII expression with and without treatment of etoposide is shown. Error bars represent SD of three replicates.

**
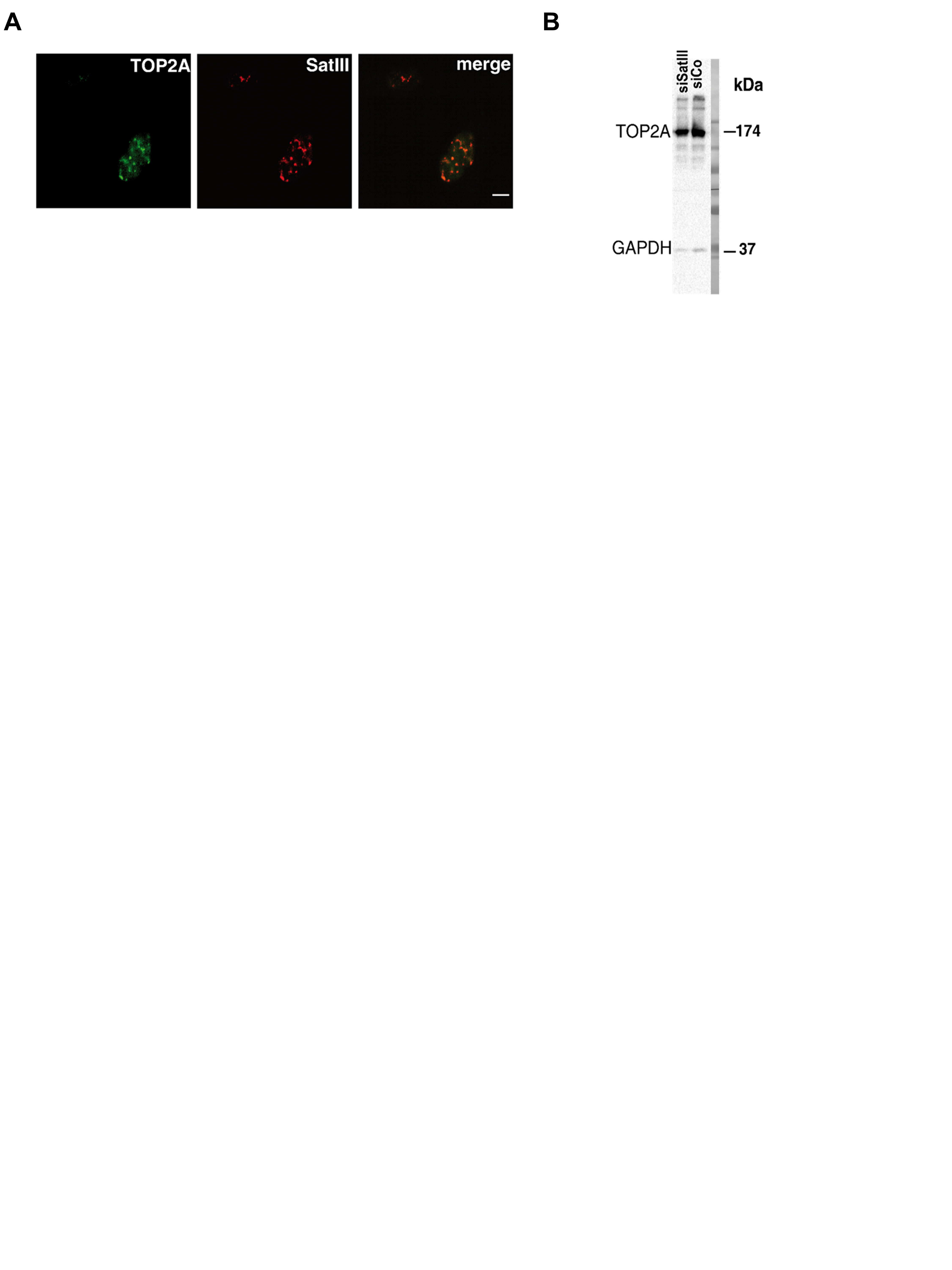
**

**Supplemental Figure 6. Related to Figure 3. SatIII co-localization with TOP2A**

**A** Representative images of co-localization of SatIII and TOP2A in U2OS cells. Cells were exposed to HS (1h at 44°C) and after 24h of recovery cultivated at 37°C. Cells were fixed and immunostained with a TOP2A antibody (green) and smFISH for SatIII (red). Scale bar, 10µm. **B** TOP2A expression is not affected by SatIII knockdown. HeLa cells were treated with either siSatIII RNA or siCo RNA. Western blotting was performed after cells were subjected to HS conditions (1h at 44°C) and 24h recovery at 37°C.


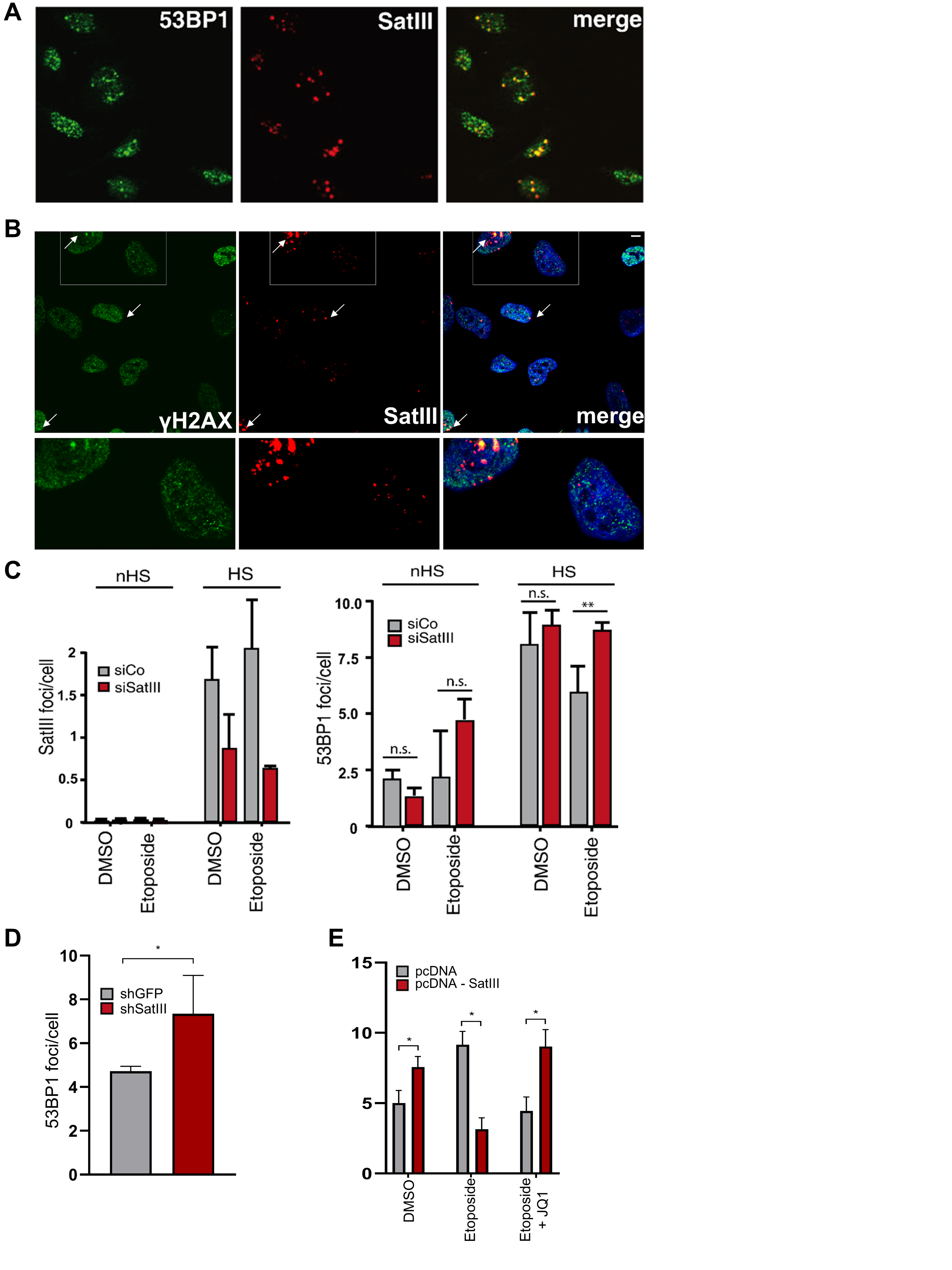


**Supplemental Figure 7. Related to Figure 3. Effects of SatIII expression on DNA damage**

**A** Representative images of immunofluorescence analyses of SatIII and 53BP1 in HeLa cells after HS (1h at 44°C). HeLa cells were exposed to HS. After 24h, cells were fixed and stained with a protein-specific 53BP1 antibody (mouse, green). Scale bar, 10µm. **B** Representative images of immunofluorescence analyses of SatIII and in HeLa cells after HS (1h at 44°C). After 24h, cells were fixed and stained with a protein-specific γH2AX antibody (mouse, green). Scale bar, 10µm. **C** Quantification of SatIII foci/cell in HeLa cells transfected with siSatIII or siCtrl and treated with etoposide. Cells were subjected to HS (1h at 44°C) and control conditions, subsequently treated with DMSO or etoposide, and fixed after 24h. SatIII staining was performed by FISH, counterstain with Hoechst stain. Quantification was acquired by HCS based microscopy, n=6. The right graph illustrates corresponding analysis of 53BP1 foci abundance under nHS and HS conditions. **D** Effects of SatIII RNA knockdown and etoposide treatment on DNA damage. HCC827 cells stably expressing shSatIII or shGFP were exposed to HS (1h at 44°C) or control conditions and treated with 20µM etoposide. Immunofluorescence and smFISH experiments were performed with staining either for 53BP1 (left) or SatIII RNA (right). Quantification was performed using an automated ImageJ pipeline. Error bars represent SD of the mean of n=5 replicates. P-values <0.01 are marked with (**). Significance was determined using two-tailed unpaired Student’s t-test. **E** Effects of SatIII RNA overexpression and etoposide and JQ1 treatment on DNA damage. HeLa cells transfected with pcDNA-SatIII or pcDNA-Ctrl were exposed to HS conditions (1h at 44°C) and treated with DMSO, etoposide or etoposide +JQ1. Immunofluorescence experiments were performed with antibodies against 53BP1. Quantifications were performed with an automated Image J pipeline. Error bars represent SD of the mean of n=5 replicates. P- values <0.001) marked with (***). Significance was determined using two-tailed unpaired Student’s t-test.





**Supplemental Figure 8. Related to Figure 4. Bromodomain inhibitor JQ1 reverts SatIII induced etoposide resistance**

**A** Representative images of smRNA FISH staining for SatIII in HeLa cells subjected to nHS and HS conditions (1h at 44°C) and with or without JQ1 treatment. Cells were treated with DMSO or JQ1 and after 24h incubation cells were fixed, immunostained, and analysed utilizing an HCS microscopy procedure. Scale bar, 50 µm. **B** qPCR experiment of HeLa cells stably overexpressing SatIII or expressing an empty control vector. Shown is the relative fold SatIII expression. **C** Cell proliferation of HeLa cells treated with 20µM etoposide or DMSO. Proliferation was measured by acquisition of images every 30 minutes over a time course of 48h. Confluency was analyzed utilizing the cell profiler software. **D** Cell proliferation assay performed as described in (C) but with a combination treatment of DMSO and 5µM JQ1. **E** Cell proliferation assay performed as described in (C) using HeLa cells either stably overexpressing SatIII RNA or expressing an empty vector control. Cells were treated with 20µM etoposide and 1µM CPI203. **F** Cell proliferation assay performed as described in (C) using H2030 cells either stably overexpressing SatIII RNA or expressing an empty vector control. Cells were treated with either 20µM etoposide and DMSO or 20µM etoposide and 1µM JQ1. **G** Cell proliferation assay performed as described in (C) using HCC827 cells either stably expressing shSatIII or expressing a shGFP control. Cells were treated with either 20µM etoposide and DMSO or 20µM etoposide and 1µM JQ1.
