# Supplementary material for: Pericentromeric Satellite III transcripts induce etoposide resistance
Source: Cell Death Dis. 2021 May 24;12(6):530. doi: 10.1038/s41419-021-03810-9 (PMC8144429; doi:10.1038/s41419-021-03810-9)
Supplement: Supplementary file 2 — Supplemental Table1 [file 41419_2021_3810_MOESM2_ESM.docx]

| **Supplementary Table 1** | Sequences of all oligonucleotides used within the study | | |  |  |  |
| --- | --- | --- | --- | --- | --- | --- |
|  |  |  |  |  |  |  |
| **Experiment** | **Gene** | **Sequence name** | **Sequence (5’-3’)** | **Source** |  |  |
| Reverse transcription | SatIII | RSM13 | ccgtaaaacgacggccagttcccttccattccattattatcc | Valgardsdottir et al. 2007 |  |  |
|  |  | FSM13 | ccgtaaaacgacggccagtcagtggaaggcattagaatcaac |  |  |  |
|  | Random | Random Hexameres | Commercially provided | Metabion |  |  |
|  |  |  |  |  |  |  |
| qPCR | SatIII | Hur98-R | aatcaacccgagtgcaatcg | Valgardsdottir et al. 2007 |  |  |
|  |  | M13 | ccgtaaaacgacggccag |  |  |  |
|  |  |  |  |  |  |  |
|  | TUBB | TUBB-fw | gctggaccgcatctcgtgta | This Paper |  |  |
|  |  | TUBBrv- | agagtccatggtcccaggtt | This Paper |  |  |
|  | NEAT1 | NEAT1-fw | gggaagggtgacattgaaaa | This Paper |  |  |
|  |  | NEAT1-rv | ctccccagcttcacttcttg | This Paper |  |  |
|  |  |  |  |  |  |  |
| Gene knockdown | SatIII | siSatIII | uggaauggaauggaauggadtdt | This Paper |  |  |
|  |  |  | uccauuccauuccauuccadtdt | This Paper |  |  |
|  |  | shSatIII | ccggtggaatggaatggaatggaaggatccatccattccat | This Paper |  |  |
|  |  |  | tccattccatttttg |  |  |  |
|  |  |  | aattcaaaaatggaatggaatggaatggatggatccttccattccattccattcca | This Paper |  |  |
|  |  | ASO-SatIII | (5’-mC*mC*mA*mU*mU*C*C*A*T*T*C*C*A*T*T*mC*mC*mA*mU*mU-3’) | Ninomiya et al. 2020 |  |  |
|  |  |  | *=Phosphorothioate bond, mN=2'-O-Methyl RNA |  |  |  |
|  | GFP | shGFP | ccgggcaagctgaccctgaagttcaggatccagaacttcagggtcagcttgctttttg | This Paper |  |  |
|  |  |  | aattcaaaaagcaagctgaccctgaagttctggatcctgaacttcagggtcagcttgc | This Paper |  |  |
|  | Ctrl | siCo | #D-001810-10-50, On-Target Plus Non-targeting Control Pool, | Dharmacon, Horizon |  |  |
|  |  | ASO Ctrl | (5’-mA*mA*mU*mG*mG*A*A*T*G*G*A*A*T*G*G*mA*mA*mU*mG*mG-3’) | Ninomiya et al. 2020 |  |  |
|  |  |  | *=Phosphorothioate bond, mN=2'-O-Methyl RNA |  |  |  |
|  |  |  |  |  |  |  |
| Methylation specific qPCR | LINE-1 | Line-1 M1-fw | cgcgagtcgaagtagggc | Iacopetta et al. 2007 |  |  |
|  |  | Line-1 UM1-fw | tgtgtgtgttgaagtagggt |  |  |  |
|  |  | Line-1 M2-rv | acccgattttccaaatacgaccg |  |  |  |
|  |  | Line-1 UM2 | acccaattttccaaatacaaccatca |  |  |  |
|  |  |  |  |  |  |  |
| Pyrosequencing | SatIII | Pyromark PCR Primer fw | ggaatggattcaacttgaatg | This Paper |  |  |
|  |  | Pyromark PCR Primer rv | ttccattccattcctatact | This Paper |  |  |
|  |  | Pyrosequencing Primer | tggaaagaatggaattaata | This Paper |  |  |
|  |  |  |  |  |  |  |
| SatIII FISH probes | SatIII | SatIII_G-rich_1 | cattccattccattccattc | This Paper |  |  |
|  |  | SatIII_G-rich_2 | cattccattgcattccattc | This Paper |  |  |
|  |  | SatIII_G-rich_3 | tgattccattccattccatt | This Paper |  |  |
|  |  | SatIII_G-rich_4 | attgcattccattccattcc | This Paper |  |  |
|  |  | SatIII_G-rich_5 | attccaatccatgccattcc | This Paper |  |  |
|  |  |  |  |  |  |  |
|  |  |  |  |  |  |  |
|  |  |  |  |  |  |  |
|  |  |  |  |  |  |  |
|  |  |  |  |  |  |  |
|  |  |  |  |  |  |  |
|  |  |  |  |  |  |  |
